# Supplementary material for: Frugivore-fruit size relationships between palms and mammals reveal past and future defaunation impacts
Source: Nat Commun. 2020 Sep 29;11:4904. doi: 10.1038/s41467-020-18530-5 (PMC7524719; doi:10.1038/s41467-020-18530-5)
Supplement: Supplementary file 1 — Supplementary Information [file 41467_2020_18530_MOESM1_ESM.pdf]

Supplementary Information

Frugivore-fruit size relationships between palms and mammals reveal past and future defaunation impacts

Lim *et al.*

This pdf file contains:

Supplementary Figures 1 - 6
Supplementary Tables 1 - 9

Supplementary Figures

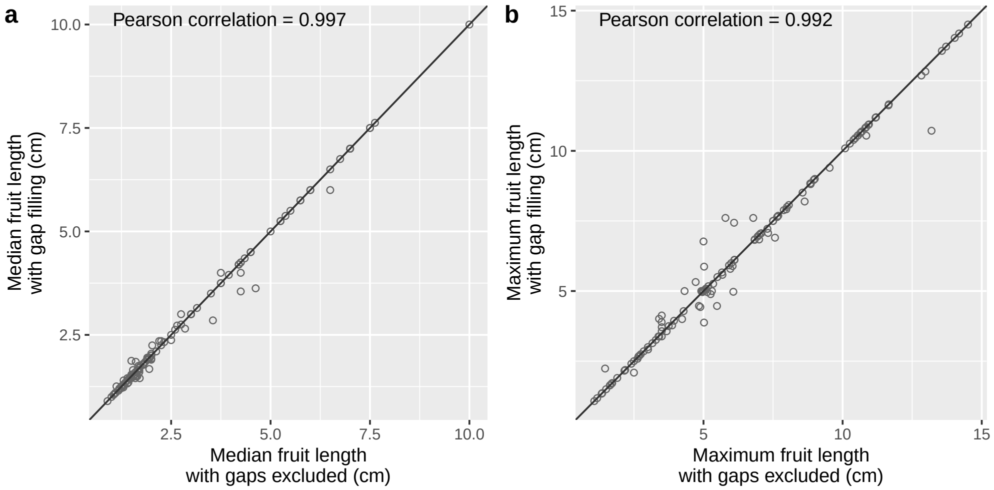

Supplementary Figure 1: **Sensitivity of botanical country median and maximum fruit size to trait imputation**. A comparison of median (a) and maximum (b) fruit length at the scale of botanical countries when species with missing data are omitted (gaps excluded) vs. imputed using genus-level means (gap filling). Black line represents the 1:1 line.

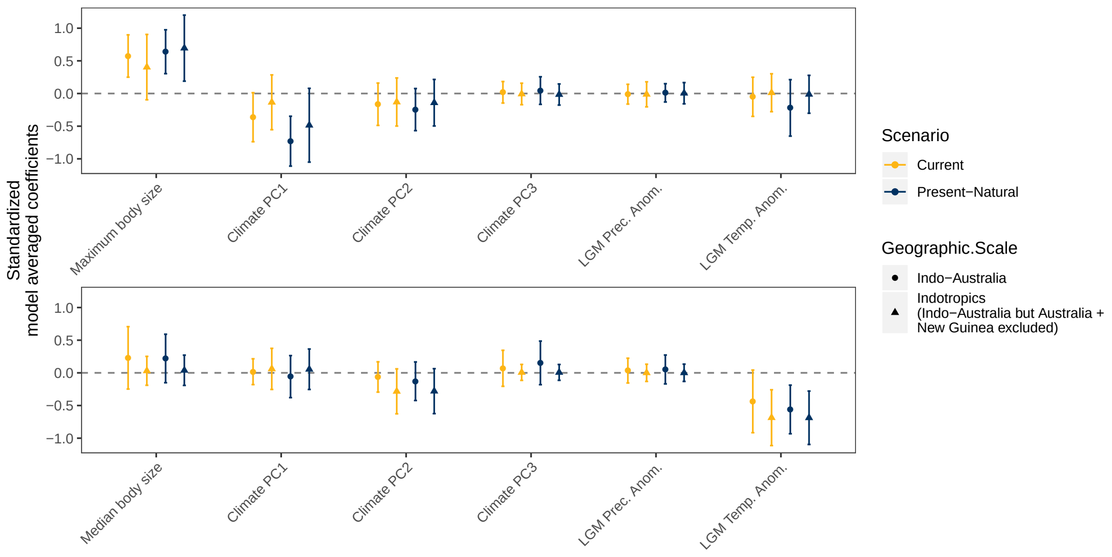

Supplementary Figure 2: **Effect of excluding Australia when analyzing patterns of median and maximum fruit size in the Indotropics under both "current" and "present-natural" scenarios.** All "maximum" values are based on 95th percentiles. Effect sizes (standardized model-averaged coefficient) are indicated by dots, and were obtained through model-averaging of ordinary least squares (OLS) models with all possible combinations of predictor variables for each scenario. Bars on each dot represent 95% confidence intervals.

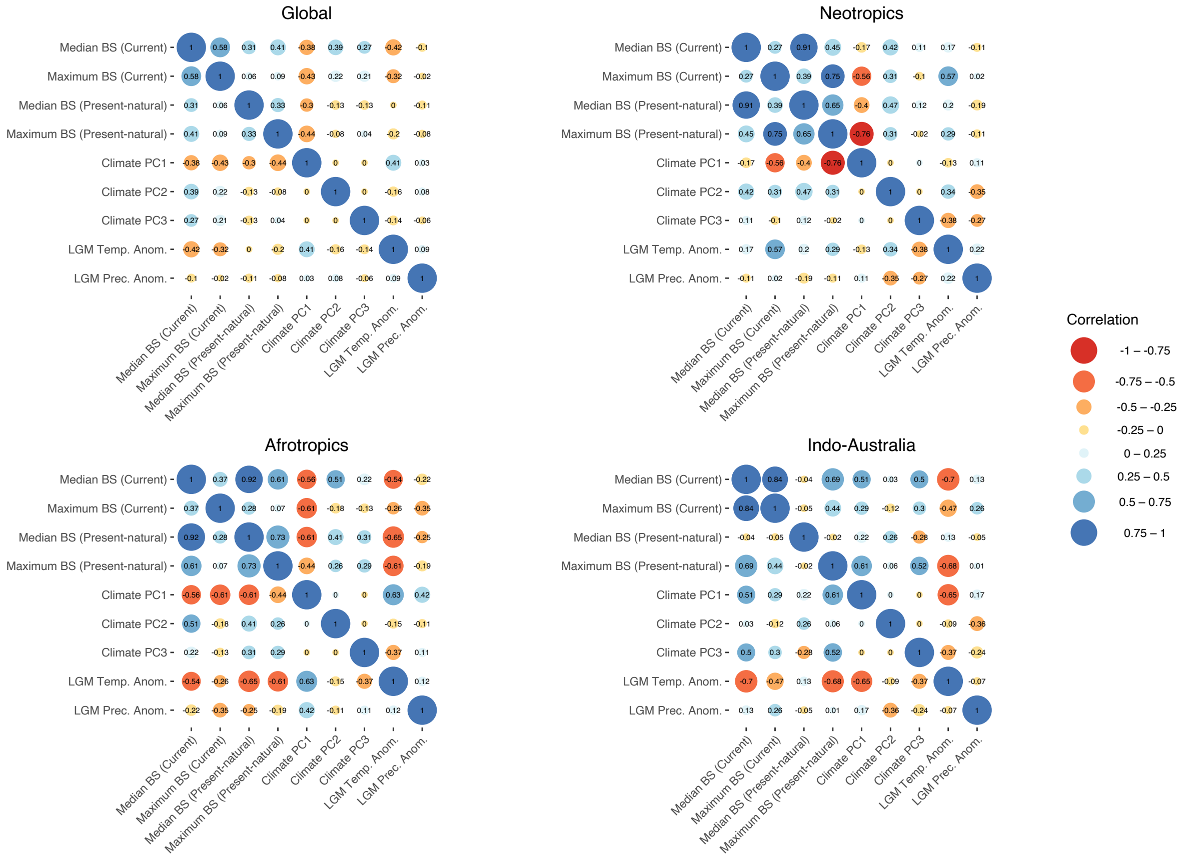

Supplementary Figure 3: **Spearman's correlation between predictor variables at global and regional scales.** Maximum (95th percentile) and median body size (BS) under either "current" and "present-natural" scenarios are used as predictor variates in separate models (see Supplementary Tables 1 – 6).

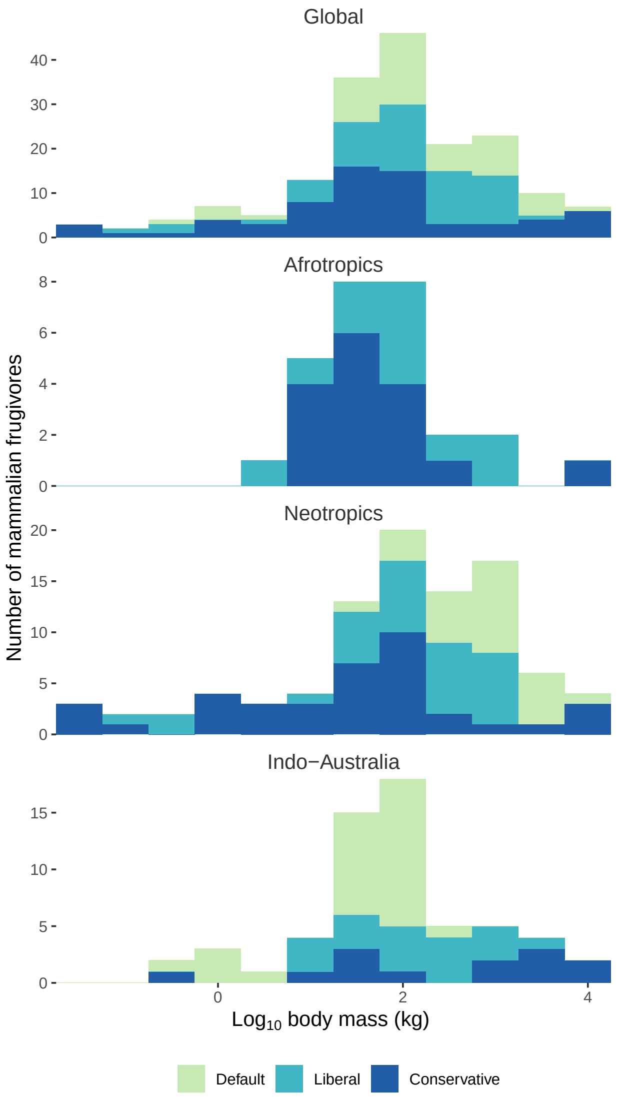

Supplementary Figure 4: **Body size distribution of putative frugivores among extinct mammals under different classifications**. Frugivore species are nested across definitions. Taxa considered under the "Default" classification are a subset of taxa considered under the "Liberal" classification, whereas taxa considered under the "Conservative" classification are a subset of taxa considered under the "Default" classification. Here, light green bars ("Default") represent taxa that are included in both the most inclusive ("Liberal") and default definition, blue-green bars ("Liberal") represent taxa that are included only under the most inclusive definition, whereas dark blue bars ("Conservative") represent taxa that are only included in the least inclusive classification (i.e., these taxa will be included under all definitions) (see Methods for how these definitions were delimited).

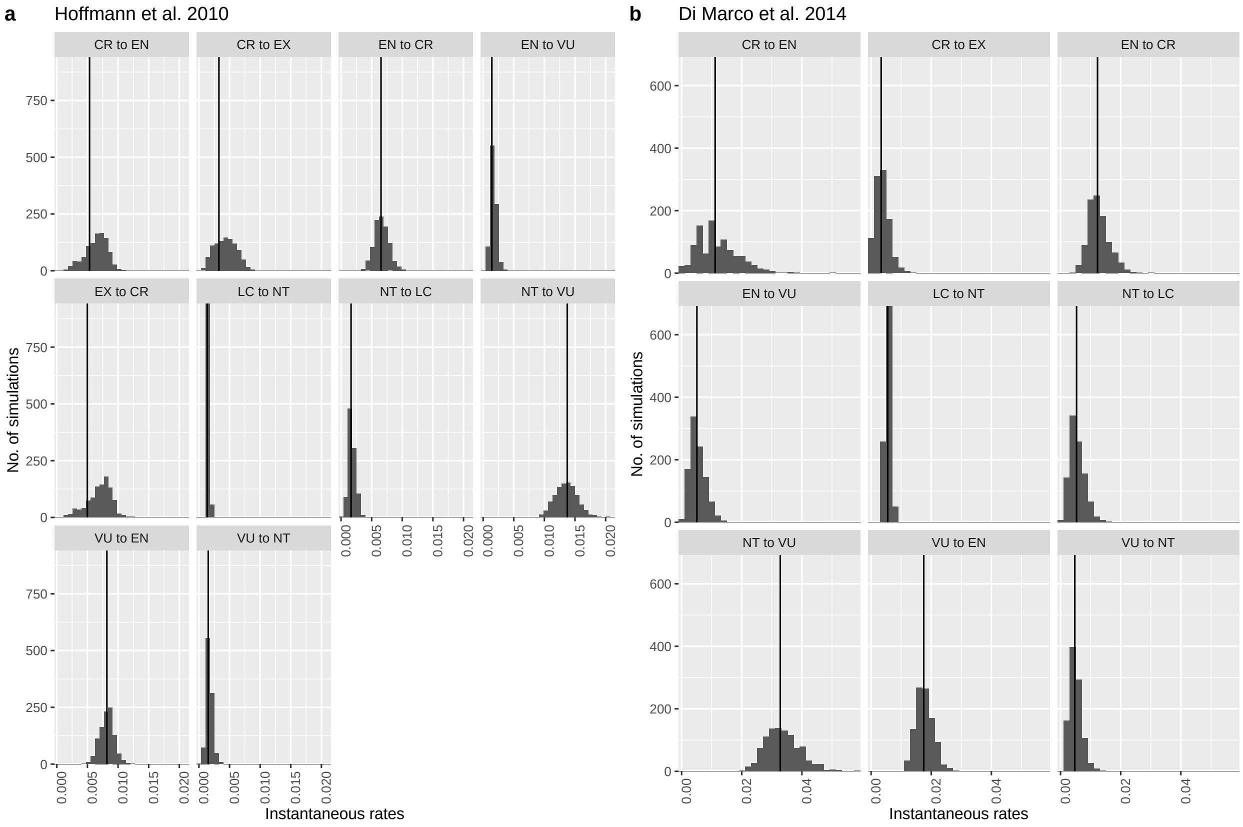

Supplementary Figure 5: **Parametric bootstrapping of CTMC rate estimates.** To evaluate the reliability of IUCN status transition rates estimated from the Hoffmann *et al.*[1] and Di Marco *et al.*[2] datasets, changes in Red List status were stochastically simulated using transition probabilities computed under empirical maximum likelihood rate estimates to generate 1000 histories, provided the same starting distribution of taxa across Red List categories and the same duration of time as each dataset. We then fit the same CTMC model to each of these 1000 randomizations and compared the empirical rates to the rates obtained from these simulations. Histograms represent the maximum likelihood rate estimates of 1000 simulations; black vertical lines represent the empirical maximum likelihood rate estimates. Most of the transition rates estimated using the CTMC model fall within the greatest density of simulated data, suggesting that rate estimates do not appear to be biased and are fairly robust given the sample size of both datasets. Estimates of the CR to EN and EX to CR transitions estimated from the Hoffmann *et al.*[1] dataset are a little lower than those derived from simulations. This may be explained by the fact that two species, *Equss ferus* and *Mustela nigripes* which were previously extinct in the wild, were downgraded to the CR and EN categories, respectively. Given the small number of such improvements from the brink of extinction, the estimation of such rates may thus have been affected by this form of stochasticity.

Supplementary Figure 6: **Potential impact of defaunation on palms**. Projected difference in true maximum fruit length given changes in true maximum present-day frugivore body size and frugivore body size of mammalian frugivore assemblages under two defaunation scenarios of varying intensity. Values can be interpreted as the degree of ecological and evolutionary change required for palm assemblages to maintain present-day relationships between maximum frugivore body size and maximum fruit size. Values are represented by a coloured circle placed at the centroid of each botanical country, with redder colours and larger circles representing greater change towards smaller fruit size.

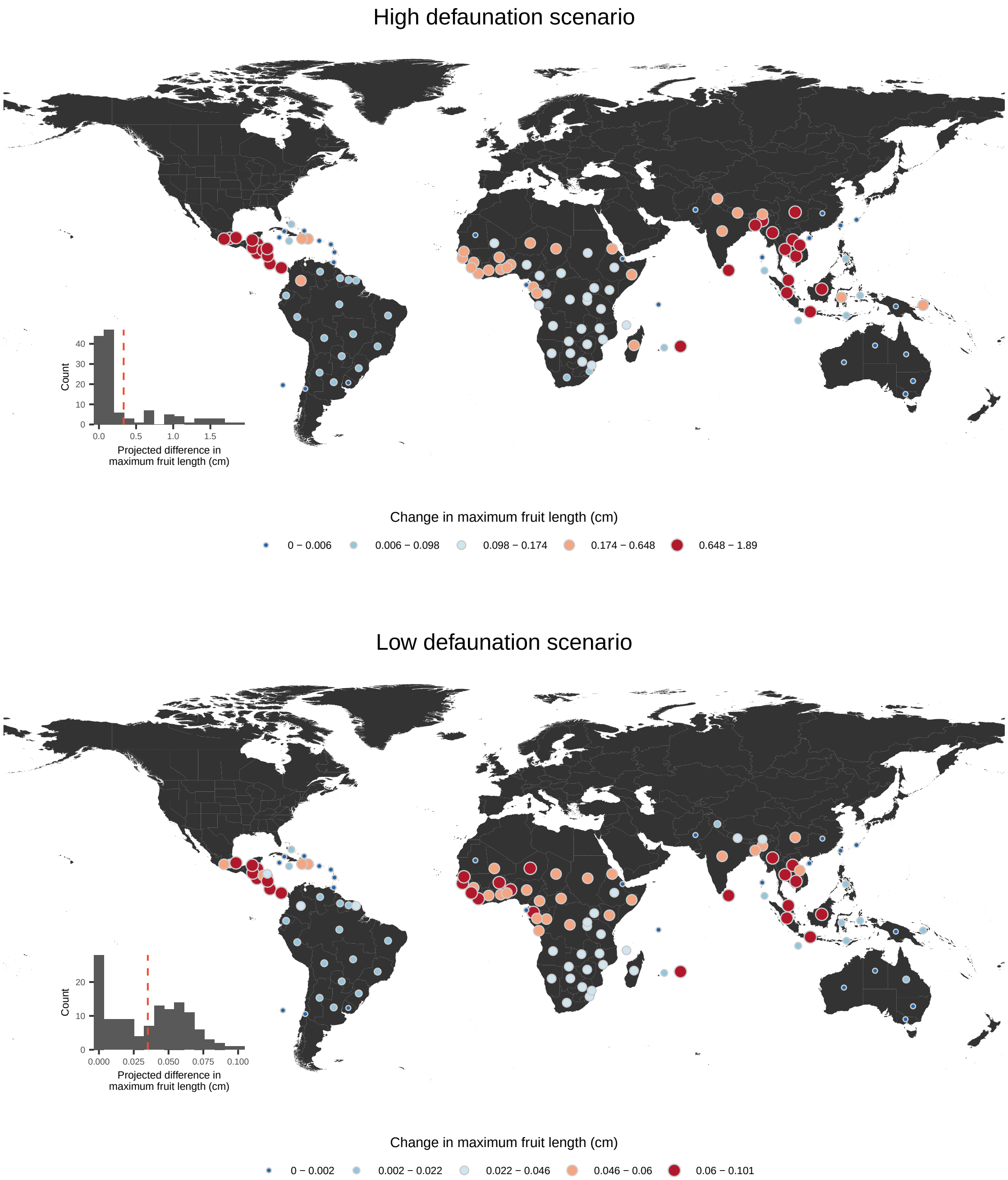

Supplementary Tables

Supplementary Table 1: Model-averaged coefficients for ordinary least square (OLS) models of maximum (95th percentile) palm fruit size with maximum (95th percentile) mammalian frugivore body size and climate as predictor variables under both "current" and "present-natural" scenarios at global and regional scales.

Geographic scale / Scenario	Variable	Model-averaged coefficient ($\bar{\beta}$)	S.E.	95% C.I.	Variance explained	R^2
**Global**						
Current	Log maximum body size	0.627	0.071	(0.486, 0.768)	0.358	0.528
	Climate PC1	0.217	0.075	(0.069, 0.365)	0.010	
	Climate PC2	0.331	0.064	(0.205, 0.458)	0.154	
	Climate PC3	-0.011	0.040	(-0.090, 0.068)	0.005	
	LGM Prec. Anom.	-0.030	0.054	(-0.136, 0.076)	0.001	
	LGM Temp. Anom.	-0.005	0.037	(-0.079, 0.069)	<0.001	
Present-Natural	Log maximum body size	0.388	0.090	(0.209, 0.566)	0.128	0.349
	Climate PC1	0.131	0.107	(-0.079, 0.341)	0.004	
	Climate PC2	0.436	0.074	(0.290, 0.582)	0.198	
	Climate PC3	0.043	0.070	(-0.095, 0.181)	0.018	
	LGM Prec. Anom.	-0.019	0.050	(-0.117, 0.080)	0.001	
	LGM Temp. Anom.	0.014	0.052	(-0.088, 0.116)	<0.001	
Present-Natural (Conservative)	Log maximum body size	0.468	0.088	(0.294, 0.641)	0.195	0.387
	Climate PC1	0.135	0.107	(-0.076, 0.345)	0.005	
	Climate PC2	0.377	0.074	(0.230, 0.524)	0.173	
	Climate PC3	0.010	0.044	(-0.077, 0.096)	0.011	
	LGM Prec. Anom.	-0.010	0.041	(-0.090, 0.071)	<0.001	
	LGM Temp. Anom.	0.089	0.095	(-0.098, 0.275)	0.003	
Present-Natural (Liberal)	Log maximum body size	0.386	0.090	(0.209, 0.563)	0.129	0.350
	Climate PC1	0.127	0.106	(-0.081, 0.335)	0.004	
	Climate PC2	0.435	0.074	(0.289, 0.581)	0.197	
	Climate PC3	0.048	0.073	(-0.095, 0.191)	0.018	
	LGM Prec. Anom.	-0.020	0.051	(-0.121, 0.081)	0.001	
	LGM Temp. Anom.	0.012	0.050	(-0.087, 0.110)	<0.001	
**Afrotropics**						
Current	Log maximum body size	0.157	0.206	(-0.250, 0.565)	0.109	0.438
	Climate PC1	0.000	0.114	(-0.226, 0.227)	0.004	
	Climate PC2	0.313	0.138	(0.039, 0.588)	0.119	
	Climate PC3	-0.392	0.221	(-0.831, 0.046)	0.146	
	LGM Prec. Anom.	-0.009	0.073	(-0.155, 0.136)	0.009	
	LGM Temp. Anom.	-0.233	0.192	(-0.613, 0.146)	0.051	
Present-Natural	Log maximum body size	-0.011	0.097	(-0.206, 0.183)	0.012	0.425
	Climate PC1	-0.027	0.111	(-0.248, 0.194)	0.017	
	Climate PC2	0.297	0.139	(0.019, 0.574)	0.115	
	Climate PC3	-0.528	0.141	(-0.810, -0.245)	0.210	
	LGM Prec. Anom.	-0.028	0.081	(-0.190, 0.135)	0.019	
	LGM Temp. Anom.	-0.305	0.184	(-0.671, 0.061)	0.051	
Present-Natural (Conservative)	Log maximum body size	-0.011	0.097	(-0.206, 0.183)	0.012	0.425
	Climate PC1	-0.027	0.111	(-0.248, 0.194)	0.017	
	Climate PC2	0.297	0.139	(0.019, 0.574)	0.115	
	Climate PC3	-0.528	0.141	(-0.810, -0.245)	0.210	
	LGM Prec. Anom.	-0.028	0.081	(-0.190, 0.135)	0.019	
	LGM Temp. Anom.	-0.305	0.184	(-0.671, 0.061)	0.051	
Present-Natural (Liberal)	Log maximum body size	-0.004	0.094	(-0.192, 0.184)	0.017	0.422
	Climate PC1	-0.026	0.110	(-0.246, 0.193)	0.016	

	Climate PC2	0.297	0.139	(0.019, 0.574)	0.114	
	Climate PC3	-0.524	0.142	(-0.809, -0.240)	0.207	
	LGM Prec. Anom.	-0.026	0.079	(-0.184, 0.132)	0.018	
	LGM Temp. Anom.	-0.300	0.184	(-0.665, 0.065)	0.050	
**Neotropics**						
Current	Log maximum body size	0.649	0.142	(0.361, 0.936)	0.292	0.453
	Climate PC1	0.372	0.143	(0.086, 0.658)	0.083	
	Climate PC2	0.002	0.058	(-0.117, 0.120)	0.001	
	Climate PC3	-0.019	0.076	(-0.172, 0.134)	0.044	
	LGM Prec. Anom.	-0.003	0.064	(-0.133, 0.127)	0.017	
	LGM Temp. Anom.	-0.010	0.075	(-0.162, 0.142)	0.016	
Present-Natural	Log maximum body size	0.675	0.135	(0.401, 0.948)	0.290	0.500
	Climate PC1	0.464	0.131	(0.199, 0.730)	0.103	
	Climate PC2	0.006	0.058	(-0.112, 0.124)	0.002	
	Climate PC3	-0.046	0.097	(-0.240, 0.148)	0.051	
	LGM Prec. Anom.	0.016	0.068	(-0.122, 0.154)	0.026	
	LGM Temp. Anom.	0.021	0.076	(-0.132, 0.173)	0.028	
Present-Natural	Log maximum body size	0.366	0.194	(-0.021, 0.752)	0.125	0.350
(Conservative)	Climate PC1	0.295	0.190	(-0.083, 0.673)	0.077	
	Climate PC2	0.007	0.076	(-0.146, 0.160)	0.002	
	Climate PC3	-0.105	0.154	(-0.412, 0.201)	0.062	
	LGM Prec. Anom.	0.046	0.111	(-0.174, 0.267)	0.033	
	LGM Temp. Anom.	0.110	0.156	(-0.201, 0.420)	0.051	
Present-Natural	Log maximum body size	0.665	0.134	(0.394, 0.936)	0.296	0.491
(Liberal)	Climate PC1	0.428	0.132	(0.161, 0.695)	0.094	
	Climate PC2	0.008	0.060	(-0.113, 0.129)	0.002	
	Climate PC3	-0.030	0.084	(-0.199, 0.138)	0.046	
	LGM Prec. Anom.	0.014	0.067	(-0.121, 0.149)	0.026	
	LGM Temp. Anom.	0.018	0.073	(-0.130, 0.165)	0.026	
**Indo-Australia**						
Current	Log maximum body size	0.574	0.160	(0.250, 0.898)	0.259	0.505
	Climate PC1	-0.363	0.187	(-0.738, 0.012)	0.110	
	Climate PC2	-0.164	0.163	(-0.488, 0.160)	0.078	
	Climate PC3	0.019	0.081	(-0.146, 0.183)	0.038	
	LGM Prec. Anom.	-0.011	0.075	(-0.162, 0.141)	0.001	
	LGM Temp. Anom.	-0.051	0.149	(-0.350, 0.249)	0.019	
Present-Natural	Log maximum body size	0.639	0.165	(0.304, 0.975)	0.195	0.565
	Climate PC1	-0.730	0.189	(-1.111, -0.348)	0.191	
	Climate PC2	-0.246	0.161	(-0.568, 0.076)	0.081	
	Climate PC3	0.045	0.105	(-0.166, 0.256)	0.061	
	LGM Prec. Anom.	0.010	0.069	(-0.130, 0.150)	0.005	
	LGM Temp. Anom.	-0.220	0.217	(-0.652, 0.212)	0.031	
Present-Natural	Log maximum body size	0.704	0.145	(0.411, 0.998)	0.321	0.584
(Conservative)	Climate PC1	-0.512	0.146	(-0.809, -0.215)	0.147	
	Climate PC2	-0.158	0.149	(-0.455, 0.139)	0.073	
	Climate PC3	-0.010	0.070	(-0.151, 0.131)	0.024	
	LGM Prec. Anom.	0.001	0.061	(-0.124, 0.126)	0.002	
	LGM Temp. Anom.	-0.040	0.119	(-0.279, 0.199)	0.018	
Present-Natural	Log maximum body size	0.584	0.168	(0.242, 0.925)	0.163	0.550
(Liberal)	Climate PC1	-0.738	0.203	(-1.148, -0.328)	0.188	
	Climate PC2	-0.281	0.163	(-0.607, 0.045)	0.086	
	Climate PC3	0.074	0.133	(-0.191, 0.339)	0.071	
	LGM Prec. Anom.	0.012	0.073	(-0.135, 0.159)	0.006	
	LGM Temp. Anom.	-0.277	0.233	(-0.741, 0.187)	0.034	

Supplementary Table 2: Model-averaged coefficients for ordinary least square (OLS) models of maximum (95th percentile) palm fruit size with maximum (95th percentile) mammalian frugivore body size and climate as predictor variables under both "current" and "present-natural" scenarios at global and regional scales. Coefficients were standardized by their partial standard deviations prior to model-averaging following Cade[3].

Geographic scale / Scenario	Variable	Model-averaged coefficient ($\bar{\beta}$)	S.E.	95% C.I.	Variance explained	R^2
**Global**						
Current	Log maximum body size	0.565	0.064	(0.439, 0.692)	0.358	0.528
	Climate PC1	0.197	0.068	(0.063, 0.332)	0.010	
	Climate PC2	0.326	0.063	(0.201, 0.451)	0.154	
	Climate PC3	-0.010	0.038	(-0.085, 0.064)	0.005	
	LGM Prec. Anom.	-0.030	0.054	(-0.137, 0.077)	0.001	
	LGM Temp. Anom.	-0.004	0.034	(-0.071, 0.063)	<0.001	
Present-Natural	Log maximum body size	0.351	0.077	(0.199, 0.504)	0.128	0.349
	Climate PC1	0.114	0.093	(-0.069, 0.297)	0.004	
	Climate PC2	0.438	0.074	(0.291, 0.585)	0.198	
	Climate PC3	0.043	0.069	(-0.093, 0.179)	0.018	
	LGM Prec. Anom.	-0.019	0.050	(-0.118, 0.080)	0.001	
	LGM Temp. Anom.	0.013	0.049	(-0.083, 0.110)	<0.001	
Present-Natural (Conservative)	Log maximum body size	0.410	0.075	(0.262, 0.558)	0.195	0.387
	Climate PC1	0.117	0.093	(-0.067, 0.301)	0.005	
	Climate PC2	0.371	0.073	(0.227, 0.515)	0.173	
	Climate PC3	0.009	0.041	(-0.073, 0.091)	0.011	
	LGM Prec. Anom.	-0.010	0.041	(-0.091, 0.071)	<0.001	
	LGM Temp. Anom.	0.082	0.087	(-0.090, 0.253)	0.003	
Present-Natural (Liberal)	Log maximum body size	0.351	0.077	(0.199, 0.503)	0.129	0.350
	Climate PC1	0.111	0.092	(-0.071, 0.293)	0.004	
	Climate PC2	0.437	0.074	(0.291, 0.584)	0.197	
	Climate PC3	0.047	0.072	(-0.094, 0.189)	0.018	
	LGM Prec. Anom.	-0.020	0.051	(-0.122, 0.081)	0.001	
	LGM Temp. Anom.	0.011	0.047	(-0.082, 0.104)	<0.001	
**Afrotropics**						
Current	Log maximum body size	0.126	0.172	(-0.214, 0.466)	0.109	0.438
	Climate PC1	-0.004	0.086	(-0.175, 0.166)	0.004	
	Climate PC2	0.317	0.140	(0.039, 0.596)	0.119	
	Climate PC3	-0.341	0.204	(-0.744, 0.063)	0.146	
	LGM Prec. Anom.	-0.009	0.067	(-0.144, 0.125)	0.009	
	LGM Temp. Anom.	-0.198	0.164	(-0.523, 0.126)	0.051	
Present-Natural	Log maximum body size	-0.006	0.071	(-0.147, 0.135)	0.012	0.425
	Climate PC1	-0.024	0.090	(-0.202, 0.155)	0.017	
	Climate PC2	0.300	0.141	(0.020, 0.581)	0.115	
	Climate PC3	-0.484	0.126	(-0.736, -0.232)	0.210	
	LGM Prec. Anom.	-0.026	0.076	(-0.178, 0.126)	0.019	
	LGM Temp. Anom.	-0.257	0.156	(-0.566, 0.053)	0.051	
Present-Natural (Conservative)	Log maximum body size	-0.006	0.071	(-0.147, 0.135)	0.012	0.425
	Climate PC1	-0.024	0.090	(-0.202, 0.155)	0.017	
	Climate PC2	0.300	0.141	(0.020, 0.581)	0.115	
	Climate PC3	-0.484	0.126	(-0.736, -0.232)	0.210	
	LGM Prec. Anom.	-0.026	0.076	(-0.178, 0.126)	0.019	
	LGM Temp. Anom.	-0.257	0.156	(-0.566, 0.053)	0.051	
Present-Natural (Liberal)	Log maximum body size	-0.001	0.070	(-0.140, 0.138)	0.017	0.422
	Climate PC1	-0.023	0.089	(-0.200, 0.154)	0.016	
	Climate PC2	0.300	0.141	(0.019, 0.581)	0.114	
	Climate PC3	-0.481	0.127	(-0.736, -0.226)	0.207	

	LGM Prec. Anom.	-0.025	0.075	(-0.174, 0.125)	0.018	
	LGM Temp. Anom.	-0.254	0.157	(-0.565, 0.057)	0.050	
**Neotropics**						
Current	Log maximum body size	0.589	0.136	(0.315, 0.862)	0.292	0.453
	Climate PC1	0.365	0.140	(0.084, 0.645)	0.083	
	Climate PC2	0.001	0.058	(-0.116, 0.119)	0.001	
	Climate PC3	-0.018	0.069	(-0.157, 0.122)	0.044	
	LGM Prec. Anom.	-0.002	0.059	(-0.121, 0.117)	0.017	
	LGM Temp. Anom.	-0.008	0.061	(-0.132, 0.115)	0.016	
Present-Natural	Log maximum body size	0.614	0.128	(0.357, 0.872)	0.290	0.500
	Climate PC1	0.439	0.124	(0.188, 0.690)	0.103	
	Climate PC2	0.006	0.058	(-0.111, 0.123)	0.002	
	Climate PC3	-0.044	0.093	(-0.230, 0.142)	0.051	
	LGM Prec. Anom.	0.016	0.066	(-0.117, 0.148)	0.026	
	LGM Temp. Anom.	0.019	0.069	(-0.120, 0.158)	0.028	
Present-Natural	Log maximum body size	0.347	0.184	(-0.019, 0.714)	0.125	0.350
(Conservative)	Climate PC1	0.286	0.184	(-0.081, 0.653)	0.077	
	Climate PC2	0.007	0.074	(-0.143, 0.156)	0.002	
	Climate PC3	-0.102	0.150	(-0.400, 0.196)	0.062	
	LGM Prec. Anom.	0.045	0.107	(-0.169, 0.259)	0.033	
	LGM Temp. Anom.	0.105	0.150	(-0.193, 0.403)	0.051	
Present-Natural	Log maximum body size	0.614	0.129	(0.353, 0.875)	0.296	0.491
(Liberal)	Climate PC1	0.412	0.128	(0.155, 0.669)	0.094	
	Climate PC2	0.008	0.060	(-0.112, 0.129)	0.002	
	Climate PC3	-0.029	0.079	(-0.187, 0.130)	0.046	
	LGM Prec. Anom.	0.014	0.064	(-0.116, 0.143)	0.026	
	LGM Temp. Anom.	0.016	0.066	(-0.117, 0.149)	0.026	
**Indo-Australia**						
Current	Log maximum body size	0.540	0.160	(0.217, 0.863)	0.259	0.505
	Climate PC1	-0.334	0.165	(-0.666, -0.002)	0.110	
	Climate PC2	-0.167	0.165	(-0.495, 0.162)	0.078	
	Climate PC3	0.017	0.075	(-0.134, 0.169)	0.038	
	LGM Prec. Anom.	-0.010	0.071	(-0.153, 0.133)	0.001	
	LGM Temp. Anom.	-0.033	0.103	(-0.240, 0.173)	0.019	
Present-Natural	Log maximum body size	0.530	0.139	(0.248, 0.812)	0.195	0.565
	Climate PC1	-0.569	0.137	(-0.848, -0.290)	0.191	
	Climate PC2	-0.253	0.166	(-0.585, 0.079)	0.081	
	Climate PC3	0.044	0.103	(-0.162, 0.249)	0.061	
	LGM Prec. Anom.	0.010	0.068	(-0.127, 0.147)	0.005	
	LGM Temp. Anom.	-0.163	0.161	(-0.484, 0.158)	0.031	
Present-Natural	Log maximum body size	0.630	0.140	(0.348, 0.913)	0.321	0.584
(Conservative)	Climate PC1	-0.462	0.128	(-0.722, -0.202)	0.147	
	Climate PC2	-0.160	0.151	(-0.462, 0.141)	0.073	
	Climate PC3	-0.009	0.060	(-0.130, 0.113)	0.024	
	LGM Prec. Anom.	0.001	0.059	(-0.120, 0.122)	0.002	
	LGM Temp. Anom.	-0.027	0.080	(-0.189, 0.135)	0.018	
Present-Natural	Log maximum body size	0.490	0.142	(0.202, 0.778)	0.163	0.550
(Liberal)	Climate PC1	-0.565	0.145	(-0.859, -0.271)	0.188	
	Climate PC2	-0.290	0.168	(-0.627, 0.047)	0.086	
	Climate PC3	0.073	0.132	(-0.189, 0.335)	0.071	
	LGM Prec. Anom.	0.012	0.071	(-0.132, 0.155)	0.006	
	LGM Temp. Anom.	-0.208	0.176	(-0.559, 0.142)	0.034	

Supplementary Table 3: Model-averaged coefficients for spatial autoregressive (SAR) models of maximum (95th percentile) palm fruit size with maximum (95th percentile) mammalian frugivore body size and climate as predictor variables under both "current" and "present-natural" scenarios at global and regional scales.

Geographic scale / Scenario	Variable	Model-averaged coefficient ($\bar{\beta}$)	S.E.	95% C.I.	Variance explained	R^2
**Global**						
Current	Log maximum body size	0.573	0.079	(0.418, 0.729)	0.193	0.649
	Climate PC1	0.216	0.107	(0.006, 0.425)	0.016	
	Climate PC2	0.235	0.075	(0.089, 0.382)	0.060	
	Climate PC3	-0.001	0.037	(-0.074, 0.073)	0.002	
	LGM Prec. Anom.	-0.007	0.036	(-0.077, 0.063)	<0.001	
	LGM Temp. Anom.	-0.069	0.084	(-0.234, 0.097)	0.004	
Present-Natural	Log maximum body size	0.377	0.073	(0.233, 0.521)	0.129	0.629
	Climate PC1	0.260	0.119	(0.027, 0.494)	0.017	
	Climate PC2	0.234	0.086	(0.066, 0.402)	0.079	
	Climate PC3	0.016	0.051	(-0.084, 0.116)	0.007	
	LGM Prec. Anom.	0.005	0.037	(-0.069, 0.078)	<0.001	
	LGM Temp. Anom.	-0.040	0.072	(-0.181, 0.101)	0.002	
Present-Natural (Conservative)	Log maximum body size	0.409	0.076	(0.260, 0.558)	0.158	0.628
	Climate PC1	0.216	0.115	(-0.010, 0.442)	0.015	
	Climate PC2	0.218	0.087	(0.047, 0.389)	0.066	
	Climate PC3	0.007	0.043	(-0.078, 0.091)	0.004	
	LGM Prec. Anom.	0.007	0.039	(-0.068, 0.083)	<0.001	
	LGM Temp. Anom.	-0.018	0.054	(-0.123, 0.087)	<0.001	
Present-Natural (Liberal)	Log maximum body size	0.393	0.073	(0.250, 0.535)	0.131	0.638
	Climate PC1	0.280	0.118	(0.049, 0.512)	0.017	
	Climate PC2	0.233	0.085	(0.067, 0.399)	0.079	
	Climate PC3	0.016	0.051	(-0.084, 0.116)	0.007	
	LGM Prec. Anom.	0.006	0.038	(-0.068, 0.080)	<0.001	
	LGM Temp. Anom.	-0.053	0.080	(-0.211, 0.104)	0.003	
**Afrotropics**						
Current	Log maximum body size	0.133	0.196	(-0.251, 0.518)	0.026	0.438
	Climate PC1	-0.001	0.110	(-0.217, 0.215)	0.007	
	Climate PC2	0.254	0.172	(-0.083, 0.590)	0.048	
	Climate PC3	-0.385	0.213	(-0.802, 0.032)	0.127	
	LGM Prec. Anom.	-0.007	0.065	(-0.135, 0.120)	0.002	
	LGM Temp. Anom.	-0.236	0.193	(-0.615, 0.142)	0.043	
Present-Natural	Log maximum body size	-0.020	0.100	(-0.215, 0.175)	0.003	0.427
	Climate PC1	-0.024	0.113	(-0.246, 0.197)	0.022	
	Climate PC2	0.224	0.173	(-0.116, 0.563)	0.051	
	Climate PC3	-0.488	0.158	(-0.798, -0.177)	0.167	
	LGM Prec. Anom.	-0.020	0.070	(-0.157, 0.118)	0.008	
	LGM Temp. Anom.	-0.297	0.191	(-0.671, 0.077)	0.053	
Present-Natural (Conservative)	Log maximum body size	-0.020	0.100	(-0.215, 0.175)	0.003	0.427
	Climate PC1	-0.024	0.113	(-0.246, 0.197)	0.022	
	Climate PC2	0.224	0.173	(-0.116, 0.563)	0.051	
	Climate PC3	-0.488	0.158	(-0.798, -0.177)	0.167	
	LGM Prec. Anom.	-0.020	0.070	(-0.157, 0.118)	0.008	
	LGM Temp. Anom.	-0.297	0.191	(-0.671, 0.077)	0.053	
Present-Natural (Liberal)	Log maximum body size	-0.009	0.091	(-0.188, 0.170)	<0.001	0.423
	Climate PC1	-0.022	0.111	(-0.239, 0.195)	0.020	
	Climate PC2	0.224	0.173	(-0.115, 0.563)	0.050	
	Climate PC3	-0.485	0.159	(-0.796, -0.173)	0.164	
	LGM Prec. Anom.	-0.018	0.068	(-0.153, 0.116)	0.006	

	LGM Temp. Anom.	-0.291	0.190	(-0.663, 0.080)	0.050	
**Neotropics**						
Current	Log maximum body size	0.569	0.144	(0.286, 0.852)	0.240	0.639
	Climate PC1	0.233	0.210	(-0.177, 0.644)	0.031	
	Climate PC2	-0.037	0.087	(-0.207, 0.133)	0.003	
	Climate PC3	-0.009	0.067	(-0.140, 0.122)	0.013	
	LGM Prec. Anom.	0.003	0.059	(-0.112, 0.119)	<0.001	
	LGM Temp. Anom.	-0.003	0.065	(-0.131, 0.125)	<0.001	
Present-Natural	Log maximum body size	0.558	0.129	(0.304, 0.811)	0.293	0.656
	Climate PC1	0.343	0.212	(-0.072, 0.759)	0.046	
	Climate PC2	-0.023	0.070	(-0.161, 0.115)	0.003	
	Climate PC3	-0.017	0.071	(-0.156, 0.121)	0.016	
	LGM Prec. Anom.	0.019	0.068	(-0.116, 0.153)	0.002	
	LGM Temp. Anom.	0.017	0.070	(-0.120, 0.153)	0.002	
Present-Natural (Conservative)	Log maximum body size	0.358	0.126	(0.111, 0.605)	0.207	0.618
	Climate PC1	0.161	0.203	(-0.236, 0.559)	0.035	
	Climate PC2	-0.026	0.078	(-0.180, 0.128)	0.003	
	Climate PC3	-0.025	0.086	(-0.193, 0.143)	0.019	
	LGM Prec. Anom.	0.019	0.075	(-0.128, 0.167)	0.004	
	LGM Temp. Anom.	0.029	0.088	(-0.143, 0.202)	0.008	
Present-Natural (Liberal)	Log maximum body size	0.578	0.123	(0.336, 0.820)	0.306	0.679
	Climate PC1	0.351	0.209	(-0.059, 0.761)	0.041	
	Climate PC2	-0.029	0.075	(-0.176, 0.119)	0.002	
	Climate PC3	-0.009	0.063	(-0.132, 0.114)	0.013	
	LGM Prec. Anom.	0.017	0.066	(-0.112, 0.146)	0.002	
	LGM Temp. Anom.	0.013	0.065	(-0.114, 0.141)	0.001	
**Indo-Australia**						
Current	Log maximum body size	0.588	0.152	(0.290, 0.885)	0.267	0.512
	Climate PC1	-0.360	0.199	(-0.750, 0.030)	0.105	
	Climate PC2	-0.151	0.158	(-0.461, 0.160)	0.070	
	Climate PC3	0.014	0.073	(-0.128, 0.157)	0.023	
	LGM Prec. Anom.	-0.009	0.070	(-0.146, 0.127)	<0.001	
	LGM Temp. Anom.	-0.056	0.149	(-0.347, 0.235)	0.018	
Present-Natural	Log maximum body size	0.643	0.151	(0.346, 0.939)	0.207	0.572
	Climate PC1	-0.750	0.189	(-1.120, -0.380)	0.188	
	Climate PC2	-0.233	0.162	(-0.550, 0.084)	0.073	
	Climate PC3	0.036	0.094	(-0.148, 0.220)	0.042	
	LGM Prec. Anom.	0.013	0.067	(-0.119, 0.144)	0.004	
	LGM Temp. Anom.	-0.228	0.218	(-0.656, 0.200)	0.029	
Present-Natural (Conservative)	Log maximum body size	0.736	0.138	(0.466, 1.006)	0.325	0.602
	Climate PC1	-0.546	0.155	(-0.850, -0.241)	0.142	
	Climate PC2	-0.147	0.146	(-0.433, 0.140)	0.066	
	Climate PC3	-0.014	0.068	(-0.148, 0.120)	0.013	
	LGM Prec. Anom.	0.006	0.059	(-0.110, 0.121)	0.001	
	LGM Temp. Anom.	-0.054	0.130	(-0.308, 0.199)	0.016	
Present-Natural (Liberal)	Log maximum body size	0.593	0.154	(0.292, 0.894)	0.175	0.559
	Climate PC1	-0.766	0.204	(-1.166, -0.366)	0.185	
	Climate PC2	-0.268	0.165	(-0.591, 0.056)	0.078	
	Climate PC3	0.058	0.118	(-0.174, 0.289)	0.051	
	LGM Prec. Anom.	0.014	0.070	(-0.123, 0.151)	0.004	
	LGM Temp. Anom.	-0.290	0.233	(-0.748, 0.167)	0.032	

Supplementary Table 4: Model-averaged coefficients for ordinary least square (OLS) models of median palm fruit size with median mammalian frugivore body size and climate as predictor variables under both "current" and "present-natural" scenarios at global and regional scales.

Geographic scale / Scenario	Variable	Model-averaged coefficient ($\bar{\beta}$)	S.E.	95% C.I.	Variance explained	R^2
**Global**						
Current	Log median body size	0.423	0.080	(0.265, 0.581)	0.244	0.528
	Climate PC1	-0.208	0.082	(-0.370, -0.046)	0.074	
	Climate PC2	0.386	0.068	(0.251, 0.521)	0.189	
	Climate PC3	-0.052	0.071	(-0.192, 0.088)	0.001	
	LGM Prec. Anom.	-0.103	0.079	(-0.259, 0.054)	0.018	
	LGM Temp. Anom.	0.048	0.071	(-0.091, 0.187)	0.001	
Present-Natural	Log median body size	0.289	0.091	(0.108, 0.469)	0.121	0.447
	Climate PC1	-0.191	0.100	(-0.387, 0.006)	0.073	
	Climate PC2	0.471	0.069	(0.334, 0.607)	0.233	
	Climate PC3	0.004	0.035	(-0.066, 0.073)	0.001	
	LGM Prec. Anom.	-0.062	0.076	(-0.212, 0.088)	0.015	
	LGM Temp. Anom.	0.009	0.044	(-0.077, 0.096)	0.005	
Present-Natural (Conservative)	Log median body size	0.342	0.082	(0.180, 0.503)	0.183	0.477
	Climate PC1	-0.234	0.083	(-0.398, -0.070)	0.081	
	Climate PC2	0.406	0.071	(0.265, 0.547)	0.198	
	Climate PC3	-0.011	0.041	(-0.093, 0.070)	<0.001	
	LGM Prec. Anom.	-0.060	0.074	(-0.205, 0.085)	0.014	
	LGM Temp. Anom.	0.053	0.076	(-0.095, 0.202)	0.001	
Present-Natural (Liberal)	Log median body size	0.348	0.091	(0.168, 0.529)	0.134	0.461
	Climate PC1	-0.127	0.104	(-0.331, 0.077)	0.065	
	Climate PC2	0.487	0.067	(0.354, 0.620)	0.242	
	Climate PC3	0.008	0.037	(-0.066, 0.081)	0.002	
	LGM Prec. Anom.	-0.058	0.074	(-0.204, 0.087)	0.015	
	LGM Temp. Anom.	0.005	0.041	(-0.076, 0.087)	0.005	
**Afrotropics**						
Current	Log median body size	-0.113	0.165	(-0.441, 0.215)	0.002	0.556
	Climate PC1	-0.630	0.171	(-0.970, -0.289)	0.308	
	Climate PC2	0.288	0.171	(-0.050, 0.627)	0.067	
	Climate PC3	-0.263	0.143	(-0.547, 0.021)	0.099	
	LGM Prec. Anom.	0.050	0.097	(-0.143, 0.243)	0.002	
	LGM Temp. Anom.	-0.068	0.136	(-0.338, 0.203)	0.078	
Present-Natural	Log median body size	-0.073	0.138	(-0.346, 0.201)	0.007	0.551
	Climate PC1	-0.609	0.169	(-0.945, -0.273)	0.300	
	Climate PC2	0.252	0.153	(-0.052, 0.555)	0.062	
	Climate PC3	-0.282	0.138	(-0.557, -0.007)	0.102	
	LGM Prec. Anom.	0.044	0.093	(-0.140, 0.229)	0.002	
	LGM Temp. Anom.	-0.079	0.145	(-0.366, 0.208)	0.079	
Present-Natural (Conservative)	Log median body size	-0.073	0.138	(-0.346, 0.201)	0.007	0.551
	Climate PC1	-0.609	0.169	(-0.945, -0.273)	0.300	
	Climate PC2	0.252	0.153	(-0.052, 0.555)	0.062	
	Climate PC3	-0.282	0.138	(-0.557, -0.007)	0.102	
	LGM Prec. Anom.	0.044	0.093	(-0.140, 0.229)	0.002	
	LGM Temp. Anom.	-0.079	0.145	(-0.366, 0.208)	0.079	
Present-Natural (Liberal)	Log median body size	-0.048	0.118	(-0.282, 0.187)	0.016	0.544
	Climate PC1	-0.600	0.171	(-0.941, -0.258)	0.290	
	Climate PC2	0.224	0.138	(-0.051, 0.499)	0.057	
	Climate PC3	-0.287	0.138	(-0.562, -0.013)	0.103	
	LGM Prec. Anom.	0.042	0.091	(-0.139, 0.223)	0.002	

	LGM Temp. Anom.	-0.082	0.148	(-0.376, 0.211)	0.077	
**Neotropics**						
Current	Log median body size	0.448	0.167	(0.114, 0.783)	0.185	0.266
	Climate PC1	-0.038	0.101	(-0.240, 0.165)	0.038	
	Climate PC2	0.017	0.082	(-0.148, 0.182)	0.018	
	Climate PC3	-0.022	0.083	(-0.189, 0.144)	0.011	
	LGM Prec. Anom.	0.007	0.071	(-0.137, 0.151)	<0.001	
	LGM Temp. Anom.	0.020	0.085	(-0.151, 0.190)	0.013	
Present-Natural	Log median body size	0.282	0.214	(-0.143, 0.708)	0.107	0.197
	Climate PC1	-0.050	0.129	(-0.309, 0.208)	0.033	
	Climate PC2	0.031	0.104	(-0.177, 0.240)	0.021	
	Climate PC3	-0.055	0.119	(-0.292, 0.182)	0.018	
	LGM Prec. Anom.	0.029	0.102	(-0.176, 0.233)	0.003	
	LGM Temp. Anom.	0.040	0.111	(-0.181, 0.262)	0.016	
Present-Natural	Log median body size	0.081	0.145	(-0.208, 0.369)	0.039	0.165
(Conservative)	Climate PC1	-0.126	0.166	(-0.457, 0.205)	0.058	
	Climate PC2	0.061	0.129	(-0.196, 0.319)	0.029	
	Climate PC3	-0.044	0.112	(-0.268, 0.180)	0.018	
	LGM Prec. Anom.	0.010	0.088	(-0.168, 0.188)	0.002	
	LGM Temp. Anom.	0.066	0.133	(-0.199, 0.331)	0.021	
Present-Natural	Log median body size	0.293	0.214	(-0.133, 0.720)	0.109	0.193
(Liberal)	Climate PC1	-0.045	0.127	(-0.299, 0.209)	0.031	
	Climate PC2	0.026	0.101	(-0.178, 0.229)	0.018	
	Climate PC3	-0.057	0.121	(-0.298, 0.184)	0.019	
	LGM Prec. Anom.	0.021	0.092	(-0.164, 0.206)	0.002	
	LGM Temp. Anom.	0.035	0.107	(-0.179, 0.250)	0.015	
**Indo-Australia**						
Current	Log median body size	0.229	0.241	(-0.248, 0.707)	0.151	0.530
	Climate PC1	0.017	0.098	(-0.180, 0.215)	0.046	
	Climate PC2	-0.063	0.116	(-0.295, 0.169)	0.018	
	Climate PC3	0.069	0.138	(-0.206, 0.345)	0.075	
	LGM Prec. Anom.	0.035	0.095	(-0.155, 0.224)	0.017	
	LGM Temp. Anom.	-0.436	0.241	(-0.916, 0.043)	0.223	
Present-Natural	Log median body size	0.220	0.186	(-0.151, 0.591)	0.115	0.600
	Climate PC1	-0.058	0.161	(-0.379, 0.264)	0.018	
	Climate PC2	-0.128	0.148	(-0.423, 0.167)	0.029	
	Climate PC3	0.152	0.167	(-0.181, 0.486)	0.119	
	LGM Prec. Anom.	0.052	0.111	(-0.169, 0.273)	0.024	
	LGM Temp. Anom.	-0.560	0.185	(-0.932, -0.188)	0.296	
Present-Natural	Log median body size	0.332	0.228	(-0.122, 0.786)	0.183	0.547
(Conservative)	Climate PC1	0.004	0.088	(-0.173, 0.182)	0.038	
	Climate PC2	-0.047	0.102	(-0.251, 0.158)	0.016	
	Climate PC3	0.039	0.114	(-0.188, 0.267)	0.066	
	LGM Prec. Anom.	0.027	0.084	(-0.142, 0.196)	0.016	
	LGM Temp. Anom.	-0.405	0.217	(-0.838, 0.028)	0.228	
Present-Natural	Log median body size	0.178	0.185	(-0.189, 0.546)	0.101	0.590
(Liberal)	Climate PC1	-0.012	0.110	(-0.233, 0.209)	0.024	
	Climate PC2	-0.129	0.151	(-0.430, 0.172)	0.032	
	Climate PC3	0.215	0.191	(-0.164, 0.595)	0.141	
	LGM Prec. Anom.	0.058	0.117	(-0.175, 0.292)	0.023	
	LGM Temp. Anom.	-0.506	0.199	(-0.905, -0.106)	0.268	

Supplementary Table 5: Model-averaged coefficients for ordinary least square (OLS) models of median palm fruit size with median mammalian frugivore body size and climate as predictor variables under both "current" and "present-natural" scenarios at global and regional scales. Coefficients were standardized by their partial standard deviations prior to model-averaging following Cade[3].

Geographic scale / Scenario	Variable	Model-averaged coefficient ($\bar{\beta}$)	S.E.	95% C.I.	Variance explained	R^2
**Global**						
Current	Log median body size	0.361	0.067	(0.228, 0.493)	0.244	0.528
	Climate PC1	-0.187	0.073	(-0.331, -0.043)	0.074	
	Climate PC2	0.367	0.065	(0.238, 0.495)	0.189	
	Climate PC3	-0.049	0.066	(-0.179, 0.082)	0.001	
	LGM Prec. Anom.	-0.103	0.080	(-0.260, 0.054)	0.018	
	LGM Temp. Anom.	0.043	0.063	(-0.082, 0.168)	0.001	
Present-Natural	Log median body size	0.248	0.086	(0.080, 0.417)	0.121	0.447
	Climate PC1	-0.161	0.085	(-0.328, 0.006)	0.073	
	Climate PC2	0.469	0.069	(0.333, 0.605)	0.233	
	Climate PC3	0.004	0.035	(-0.066, 0.073)	0.001	
	LGM Prec. Anom.	-0.061	0.075	(-0.208, 0.086)	0.015	
	LGM Temp. Anom.	0.008	0.040	(-0.071, 0.087)	0.005	
Present-Natural (Conservative)	Log median body size	0.291	0.071	(0.151, 0.430)	0.183	0.477
	Climate PC1	-0.209	0.073	(-0.354, -0.064)	0.081	
	Climate PC2	0.385	0.067	(0.252, 0.518)	0.198	
	Climate PC3	-0.011	0.040	(-0.089, 0.067)	<0.001	
	LGM Prec. Anom.	-0.059	0.072	(-0.202, 0.084)	0.014	
	LGM Temp. Anom.	0.048	0.067	(-0.085, 0.180)	0.001	
Present-Natural (Liberal)	Log median body size	0.304	0.096	(0.115, 0.494)	0.134	0.461
	Climate PC1	-0.104	0.084	(-0.270, 0.063)	0.065	
	Climate PC2	0.489	0.068	(0.355, 0.623)	0.242	
	Climate PC3	0.008	0.037	(-0.066, 0.082)	0.002	
	LGM Prec. Anom.	-0.058	0.073	(-0.200, 0.085)	0.015	
	LGM Temp. Anom.	0.005	0.038	(-0.070, 0.080)	0.005	
**Afrotropics**						
Current	Log median body size	-0.077	0.115	(-0.305, 0.151)	0.002	0.556
	Climate PC1	-0.525	0.161	(-0.843, -0.206)	0.308	
	Climate PC2	0.249	0.137	(-0.024, 0.522)	0.067	
	Climate PC3	-0.253	0.135	(-0.521, 0.015)	0.099	
	LGM Prec. Anom.	0.046	0.089	(-0.132, 0.224)	0.002	
	LGM Temp. Anom.	-0.047	0.097	(-0.239, 0.145)	0.078	
Present-Natural	Log median body size	-0.051	0.097	(-0.243, 0.142)	0.007	0.551
	Climate PC1	-0.505	0.160	(-0.822, -0.188)	0.300	
	Climate PC2	0.233	0.136	(-0.038, 0.504)	0.062	
	Climate PC3	-0.272	0.130	(-0.532, -0.013)	0.102	
	LGM Prec. Anom.	0.041	0.085	(-0.129, 0.211)	0.002	
	LGM Temp. Anom.	-0.055	0.102	(-0.258, 0.148)	0.079	
Present-Natural (Conservative)	Log median body size	-0.051	0.097	(-0.243, 0.142)	0.007	0.551
	Climate PC1	-0.505	0.160	(-0.822, -0.188)	0.300	
	Climate PC2	0.233	0.136	(-0.038, 0.504)	0.062	
	Climate PC3	-0.272	0.130	(-0.532, -0.013)	0.102	
	LGM Prec. Anom.	0.041	0.085	(-0.129, 0.211)	0.002	
	LGM Temp. Anom.	-0.055	0.102	(-0.258, 0.148)	0.079	
Present-Natural (Liberal)	Log median body size	-0.032	0.080	(-0.191, 0.127)	0.016	0.544
	Climate PC1	-0.489	0.162	(-0.811, -0.168)	0.290	
	Climate PC2	0.222	0.136	(-0.049, 0.493)	0.057	
	Climate PC3	-0.277	0.130	(-0.535, -0.019)	0.103	

	LGM Prec. Anom.	0.039	0.084	(-0.128, 0.206)	0.002	
	LGM Temp. Anom.	-0.057	0.104	(-0.263, 0.149)	0.077	
**Neotropics**						
Current	Log median body size	0.438	0.164	(0.108, 0.767)	0.185	0.266
	Climate PC1	-0.036	0.098	(-0.233, 0.160)	0.038	
	Climate PC2	0.017	0.078	(-0.141, 0.174)	0.018	
	Climate PC3	-0.022	0.082	(-0.188, 0.143)	0.011	
	LGM Prec. Anom.	0.007	0.070	(-0.135, 0.148)	$<$0.001	
	LGM Temp. Anom.	0.019	0.080	(-0.142, 0.180)	0.013	
Present-Natural	Log median body size	0.264	0.202	(-0.136, 0.665)	0.107	0.197
	Climate PC1	-0.048	0.123	(-0.294, 0.197)	0.033	
	Climate PC2	0.030	0.099	(-0.168, 0.228)	0.021	
	Climate PC3	-0.054	0.118	(-0.289, 0.181)	0.018	
	LGM Prec. Anom.	0.027	0.095	(-0.164, 0.217)	0.003	
	LGM Temp. Anom.	0.039	0.106	(-0.173, 0.251)	0.016	
Present-Natural	Log median body size	0.079	0.140	(-0.201, 0.358)	0.039	0.165
(Conservative)	Climate PC1	-0.127	0.167	(-0.459, 0.206)	0.058	
	Climate PC2	0.060	0.126	(-0.191, 0.310)	0.029	
	Climate PC3	-0.043	0.110	(-0.264, 0.178)	0.018	
	LGM Prec. Anom.	0.009	0.083	(-0.158, 0.177)	0.002	
	LGM Temp. Anom.	0.064	0.129	(-0.193, 0.322)	0.021	
Present-Natural	Log median body size	0.274	0.202	(-0.128, 0.675)	0.109	0.193
(Liberal)	Climate PC1	-0.043	0.120	(-0.283, 0.197)	0.031	
	Climate PC2	0.024	0.095	(-0.166, 0.215)	0.018	
	Climate PC3	-0.057	0.119	(-0.295, 0.182)	0.019	
	LGM Prec. Anom.	0.020	0.087	(-0.155, 0.196)	0.002	
	LGM Temp. Anom.	0.034	0.102	(-0.170, 0.237)	0.015	
**Indo-Australia**						
Current	Log median body size	0.181	0.202	(-0.218, 0.580)	0.151	0.530
	Climate PC1	0.015	0.083	(-0.152, 0.182)	0.046	
	Climate PC2	-0.063	0.116	(-0.295, 0.169)	0.018	
	Climate PC3	0.062	0.123	(-0.183, 0.307)	0.075	
	LGM Prec. Anom.	0.034	0.092	(-0.151, 0.219)	0.017	
	LGM Temp. Anom.	-0.364	0.223	(-0.807, 0.078)	0.223	
Present-Natural	Log median body size	0.199	0.165	(-0.130, 0.528)	0.115	0.600
	Climate PC1	-0.036	0.107	(-0.250, 0.178)	0.018	
	Climate PC2	-0.130	0.151	(-0.430, 0.170)	0.029	
	Climate PC3	0.143	0.157	(-0.170, 0.456)	0.119	
	LGM Prec. Anom.	0.051	0.109	(-0.166, 0.268)	0.024	
	LGM Temp. Anom.	-0.491	0.165	(-0.822, -0.159)	0.296	
Present-Natural	Log median body size	0.267	0.194	(-0.118, 0.653)	0.183	0.547
(Conservative)	Climate PC1	0.004	0.072	(-0.142, 0.150)	0.038	
	Climate PC2	-0.046	0.102	(-0.250, 0.157)	0.016	
	Climate PC3	0.035	0.100	(-0.164, 0.234)	0.066	
	LGM Prec. Anom.	0.027	0.083	(-0.139, 0.193)	0.016	
	LGM Temp. Anom.	-0.333	0.192	(-0.716, 0.050)	0.228	
Present-Natural	Log median body size	0.159	0.165	(-0.169, 0.487)	0.101	0.590
(Liberal)	Climate PC1	-0.007	0.081	(-0.169, 0.156)	0.024	
	Climate PC2	-0.130	0.152	(-0.433, 0.173)	0.032	
	Climate PC3	0.197	0.176	(-0.153, 0.547)	0.141	
	LGM Prec. Anom.	0.057	0.114	(-0.171, 0.285)	0.023	
	LGM Temp. Anom.	-0.445	0.190	(-0.824, -0.067)	0.268	

Supplementary Table 6: Model-averaged coefficients for spatial autoregressive (SAR) models of median palm fruit size with median mammalian frugivore body size and climate as predictor variables under both "current" and "present-natural" scenarios at global and regional scales.

Geographic scale / Scenario	Variable	Model-averaged coefficient ($\bar{\beta}$)	S.E.	95% C.I.	Variance explained	R^2
**Global**						
Current	Log median body size	0.019	0.056	(-0.090, 0.128)	0.023	0.774
	Climate PC1	-0.146	0.109	(-0.359, 0.068)	0.041	
	Climate PC2	0.135	0.081	(-0.024, 0.294)	0.057	
	Climate PC3	0.077	0.082	(-0.084, 0.238)	0.007	
	LGM Prec. Anom.	-0.004	0.036	(-0.074, 0.065)	0.002	
	LGM Temp. Anom.	-0.064	0.078	(-0.217, 0.089)	0.012	
Present-Natural	Log median body size	0.007	0.039	(-0.068, 0.083)	0.020	0.775
	Climate PC1	-0.144	0.110	(-0.360, 0.073)	0.037	
	Climate PC2	0.136	0.081	(-0.023, 0.294)	0.061	
	Climate PC3	0.079	0.083	(-0.082, 0.241)	0.009	
	LGM Prec. Anom.	-0.004	0.035	(-0.073, 0.066)	0.002	
	LGM Temp. Anom.	-0.066	0.079	(-0.221, 0.088)	0.013	
Present-Natural (Conservative)	Log median body size	0.002	0.039	(-0.076, 0.079)	0.016	0.776
	Climate PC1	-0.147	0.110	(-0.362, 0.068)	0.043	
	Climate PC2	0.135	0.081	(-0.024, 0.293)	0.059	
	Climate PC3	0.081	0.083	(-0.082, 0.244)	0.008	
	LGM Prec. Anom.	-0.004	0.036	(-0.074, 0.066)	0.002	
	LGM Temp. Anom.	-0.066	0.079	(-0.220, 0.089)	0.012	
Present-Natural (Liberal)	Log median body size	0.009	0.039	(-0.068, 0.086)	0.022	0.775
	Climate PC1	-0.144	0.110	(-0.359, 0.072)	0.035	
	Climate PC2	0.136	0.081	(-0.022, 0.295)	0.063	
	Climate PC3	0.080	0.082	(-0.082, 0.241)	0.009	
	LGM Prec. Anom.	-0.004	0.035	(-0.073, 0.066)	0.002	
	LGM Temp. Anom.	-0.066	0.079	(-0.219, 0.088)	0.013	
**Afrotropics**						
Current	Log median body size	-0.103	0.156	(-0.408, 0.203)	0.002	0.558
	Climate PC1	-0.624	0.170	(-0.958, -0.291)	0.214	
	Climate PC2	0.262	0.179	(-0.088, 0.613)	0.041	
	Climate PC3	-0.254	0.146	(-0.540, 0.033)	0.078	
	LGM Prec. Anom.	0.048	0.092	(-0.134, 0.229)	0.002	
	LGM Temp. Anom.	-0.065	0.132	(-0.324, 0.195)	0.054	
Present-Natural	Log median body size	-0.066	0.128	(-0.317, 0.185)	$<$0.001	0.553
	Climate PC1	-0.605	0.168	(-0.935, -0.275)	0.212	
	Climate PC2	0.225	0.161	(-0.090, 0.540)	0.037	
	Climate PC3	-0.267	0.145	(-0.552, 0.018)	0.082	
	LGM Prec. Anom.	0.043	0.089	(-0.131, 0.217)	0.001	
	LGM Temp. Anom.	-0.074	0.140	(-0.348, 0.200)	0.058	
Present-Natural (Conservative)	Log median body size	-0.066	0.128	(-0.317, 0.185)	$<$0.001	0.553
	Climate PC1	-0.605	0.168	(-0.935, -0.275)	0.212	
	Climate PC2	0.225	0.161	(-0.090, 0.540)	0.037	
	Climate PC3	-0.267	0.145	(-0.552, 0.018)	0.082	
	LGM Prec. Anom.	0.043	0.089	(-0.131, 0.217)	0.001	
	LGM Temp. Anom.	-0.074	0.140	(-0.348, 0.200)	0.058	
Present-Natural (Liberal)	Log median body size	-0.045	0.111	(-0.262, 0.173)	0.001	0.548
	Climate PC1	-0.597	0.172	(-0.933, -0.261)	0.208	
	Climate PC2	0.199	0.146	(-0.087, 0.485)	0.030	
	Climate PC3	-0.272	0.145	(-0.557, 0.013)	0.083	
	LGM Prec. Anom.	0.041	0.087	(-0.130, 0.212)	0.001	

	LGM Temp. Anom.	-0.077	0.143	(-0.357, 0.203)	0.057	
**Neotropics**						
Current	Log median body size	0.045	0.097	(-0.144, 0.235)	0.090	0.635
	Climate PC1	-0.064	0.140	(-0.338, 0.211)	0.034	
	Climate PC2	-0.012	0.068	(-0.146, 0.122)	0.001	
	Climate PC3	-0.094	0.147	(-0.383, 0.195)	0.026	
	LGM Prec. Anom.	-0.002	0.069	(-0.138, 0.133)	0.001	
	LGM Temp. Anom.	-0.098	0.145	(-0.382, 0.185)	0.011	
Present-Natural	Log median body size	0.021	0.075	(-0.126, 0.168)	0.064	0.636
	Climate PC1	-0.062	0.140	(-0.338, 0.213)	0.028	
	Climate PC2	-0.011	0.068	(-0.144, 0.122)	0.001	
	Climate PC3	-0.094	0.147	(-0.383, 0.195)	0.034	
	LGM Prec. Anom.	-0.004	0.069	(-0.140, 0.132)	$<$0.001	
	LGM Temp. Anom.	-0.104	0.148	(-0.393, 0.185)	0.011	
Present-Natural (Conservative)	Log median body size	0.005	0.053	(-0.100, 0.110)	0.023	0.635
	Climate PC1	-0.066	0.143	(-0.345, 0.213)	0.046	
	Climate PC2	-0.011	0.068	(-0.143, 0.122)	0.003	
	Climate PC3	-0.096	0.149	(-0.387, 0.195)	0.034	
	LGM Prec. Anom.	-0.005	0.070	(-0.141, 0.131)	$<$0.001	
	LGM Temp. Anom.	-0.107	0.149	(-0.399, 0.185)	0.008	
Present-Natural (Liberal)	Log median body size	-0.004	0.066	(-0.134, 0.125)	0.034	0.641
	Climate PC1	-0.069	0.147	(-0.357, 0.219)	0.033	
	Climate PC2	-0.010	0.068	(-0.143, 0.123)	0.001	
	Climate PC3	-0.096	0.149	(-0.388, 0.196)	0.033	
	LGM Prec. Anom.	-0.006	0.070	(-0.143, 0.131)	$<$0.001	
	LGM Temp. Anom.	-0.108	0.150	(-0.401, 0.185)	0.009	
**Indo-Australia**						
Current	Log median body size	0.320	0.234	(-0.139, 0.778)	0.035	0.595
	Climate PC1	0.011	0.074	(-0.134, 0.156)	0.012	
	Climate PC2	-0.044	0.092	(-0.225, 0.137)	0.006	
	Climate PC3	0.063	0.126	(-0.185, 0.311)	0.020	
	LGM Prec. Anom.	0.048	0.094	(-0.136, 0.232)	0.008	
	LGM Temp. Anom.	-0.449	0.217	(-0.874, -0.023)	0.138	
Present-Natural	Log median body size	0.266	0.153	(-0.033, 0.566)	0.079	0.693
	Climate PC1	-0.089	0.161	(-0.404, 0.225)	0.001	
	Climate PC2	-0.083	0.117	(-0.313, 0.147)	0.010	
	Climate PC3	0.250	0.156	(-0.057, 0.556)	0.027	
	LGM Prec. Anom.	0.141	0.136	(-0.125, 0.408)	0.010	
	LGM Temp. Anom.	-0.586	0.174	(-0.927, -0.245)	0.163	
Present-Natural (Conservative)	Log median body size	0.454	0.139	(0.183, 0.726)	0.047	0.657
	Climate PC1	-0.008	0.055	(-0.116, 0.100)	0.010	
	Climate PC2	-0.016	0.056	(-0.126, 0.095)	0.005	
	Climate PC3	0.011	0.067	(-0.119, 0.142)	0.016	
	LGM Prec. Anom.	0.035	0.072	(-0.107, 0.176)	0.008	
	LGM Temp. Anom.	-0.433	0.142	(-0.713, -0.154)	0.137	
Present-Natural (Liberal)	Log median body size	0.278	0.160	(-0.036, 0.592)	0.068	0.710
	Climate PC1	-0.059	0.128	(-0.310, 0.193)	0.002	
	Climate PC2	-0.098	0.122	(-0.336, 0.141)	0.012	
	Climate PC3	0.332	0.153	(0.033, 0.631)	0.037	
	LGM Prec. Anom.	0.156	0.137	(-0.113, 0.424)	0.010	
	LGM Temp. Anom.	-0.515	0.166	(-0.841, -0.189)	0.145	

Supplementary Table 7: **Potential Pleistocene frugivores**. List of all Pleistocene mammals in the PHYLACINE dataset[4] and their inclusion in the present-natural scenario under our default (D), "Conservative" (C) and "Liberal" (L) classifications (see Methods). Prop_{frug} columns show the proportion of extant species at the family or order level that are considered frugivorous based on the MammalDiet dataset[5].

Order	Family	Binomial name	Prop_{frug} (Family-level)	Prop_{frug} (Order-level)	L	D	C
Afrosoricida	Tenrecidae	Microgale_macpheei	0	0	N	N	N
Afrosoricida	Tenrecidae	Plesiorycteropus_madagascariensis	0	0	N	N	N
Carnivora	Canidae	Canis_dirus	0.417	0.373	N	N	N
Carnivora	Canidae	Cynotherium_sardous	0.417	0.373	N	N	N
Carnivora	Canidae	Protocyon_troglodytes	0.417	0.373	N	N	N
Carnivora	Canidae	Theriodictis_tarijensis	0.417	0.373	N	N	N
Carnivora	Felidae	Homotherium_latidens	0.028	0.373	N	N	N
Carnivora	Felidae	Homotherium_serum	0.028	0.373	N	N	N
Carnivora	Felidae	Leopardus_amnicola	0.028	0.373	N	N	N
Carnivora	Felidae	Miracinonyx_trumani	0.028	0.373	N	N	N
Carnivora	Felidae	Panthera_atrox	0.028	0.373	N	N	N
Carnivora	Felidae	Panthera_spelaea	0.028	0.373	N	N	N
Carnivora	Felidae	Smilodon_fatalis	0.028	0.373	N	N	N
Carnivora	Felidae	Smilodon_populator	0.028	0.373	N	N	N
Carnivora	Mephitidae	Brachyprotoma_obtusata	0.5	0.373	N	N	N
Carnivora	Ursidae	Arctodus_simus	0.75	0.373	N	N	N
Carnivora	Ursidae	Arctotherium_tarijense	0.75	0.373	Y	Y	Y
Carnivora	Ursidae	Arctotherium_wingei	0.75	0.373	Y	Y	Y
Carnivora	Ursidae	Tremarctos_floridanus	0.75	0.373	Y	Y	Y
Carnivora	Ursidae	Ursus_spelaeus	0.75	0.373	Y	Y	Y
Cetartiodactyla	Antilocapridae	Capromeryx_minor	0	0.332	Y	N	N
Cetartiodactyla	Antilocapridae	Stockoceros_conklingi	0	0.332	Y	N	N
Cetartiodactyla	Antilocapridae	Tetrameryx_shuleri	0	0.332	Y	N	N
Cetartiodactyla	Bovidae	Antidorcas_australis	0.25	0.332	Y	N	N
Cetartiodactyla	Bovidae	Antidorcas_bondi	0.25	0.332	Y	N	N
Cetartiodactyla	Bovidae	Bootherium_bombifrons	0.25	0.332	Y	N	N
Cetartiodactyla	Bovidae	Bubalus_grovesi	0.25	0.332	Y	N	N
Cetartiodactyla	Bovidae	Bubalus_palaeokerabau	0.25	0.332	Y	N	N
Cetartiodactyla	Bovidae	CapriniGen_spA	0.25	0.332	Y	N	N
Cetartiodactyla	Bovidae	Damaliscus_hypsodon	0.25	0.332	Y	N	N
Cetartiodactyla	Bovidae	Damaliscus_niro	0.25	0.332	Y	N	N
Cetartiodactyla	Bovidae	Euceratherium_collinum	0.25	0.332	Y	N	N
Cetartiodactyla	Bovidae	Gazella_atlantica	0.25	0.332	Y	N	N
Cetartiodactyla	Bovidae	Gazella_tingitana	0.25	0.332	Y	N	N
Cetartiodactyla	Bovidae	Hemitragus_cedrensis	0.25	0.332	Y	N	N
Cetartiodactyla	Bovidae	Megalotragus_priscus	0.25	0.332	Y	N	N
Cetartiodactyla	Bovidae	Megalovis_guangxiensis	0.25	0.332	Y	N	N
Cetartiodactyla	Bovidae	Myotragus_balearicus	0.25	0.332	Y	N	N
Cetartiodactyla	Bovidae	Oreamnos_harringtoni	0.25	0.332	Y	N	N
Cetartiodactyla	Bovidae	Pelorovis_antiquus	0.25	0.332	Y	N	N
Cetartiodactyla	Bovidae	Rusingoryx_atopocranion	0.25	0.332	Y	N	N
Cetartiodactyla	Bovidae	Sivacobus_sankaliai	0.25	0.332	Y	N	N
Cetartiodactyla	Bovidae	Soergelia_minor	0.25	0.332	Y	N	N
Cetartiodactyla	Bovidae	Spirocerus_kiakhtensis	0.25	0.332	Y	N	N
Cetartiodactyla	Camelidae	Camelops_hesternus	0	0.332	Y	N	N
Cetartiodactyla	Camelidae	Camelus_dromedarius	0	0.332	Y	N	N
Cetartiodactyla	Camelidae	Hemiauchenia_macrocephala	0	0.332	Y	N	N

Order	Family	Binomial name	Prop$_{frug}$ (Family-level)	Prop$_{frug}$ (Order-level)	L	D	C
Cetartiodactyla	Camelidae	Hemiauchenia_paradoxa	0	0.332	Y	N	N
Cetartiodactyla	Camelidae	Palaeolama_major	0	0.332	Y	N	N
Cetartiodactyla	Camelidae	Palaeolama_mirifica	0	0.332	Y	N	N
Cetartiodactyla	Camelidae	Palaeolama_weddeli	0	0.332	Y	N	N
Cetartiodactyla	Cervidae	Agalmaceros_blicki	0.273	0.332	Y	N	N
Cetartiodactyla	Cervidae	Antifer_ultra	0.273	0.332	Y	N	N
Cetartiodactyla	Cervidae	Candiacervus_cretensis	0.273	0.332	Y	N	N
Cetartiodactyla	Cervidae	Candiacervus_dorothensis	0.273	0.332	Y	N	N
Cetartiodactyla	Cervidae	Candiacervus_major	0.273	0.332	Y	N	N
Cetartiodactyla	Cervidae	Candiacervus_rethymnensis	0.273	0.332	Y	N	N
Cetartiodactyla	Cervidae	Candiacervus_ropalophorus	0.273	0.332	Y	N	N
Cetartiodactyla	Cervidae	Candiacervus_spII	0.273	0.332	Y	N	N
Cetartiodactyla	Cervidae	Capreolus_miyakoensis	0.273	0.332	Y	N	N
Cetartiodactyla	Cervidae	Cervalces_scotti	0.273	0.332	Y	N	N
Cetartiodactyla	Cervidae	Cervus_astylodon	0.273	0.332	Y	N	N
Cetartiodactyla	Cervidae	Dicroceros_spA	0.273	0.332	Y	N	N
Cetartiodactyla	Cervidae	Haploidoceros_mediterraneus	0.273	0.332	Y	N	N
Cetartiodactyla	Cervidae	Megaceroides_algericus	0.273	0.332	Y	N	N
Cetartiodactyla	Cervidae	Megaloceros_giganteus	0.273	0.332	Y	N	N
Cetartiodactyla	Cervidae	Morenelaphus_brachyceros	0.273	0.332	Y	N	N
Cetartiodactyla	Cervidae	Morenelaphus_lujanensis	0.273	0.332	Y	N	N
Cetartiodactyla	Cervidae	Navahoceros_fricki	0.273	0.332	Y	N	N
Cetartiodactyla	Cervidae	Paraceros_fragilis	0.273	0.332	Y	N	N
Cetartiodactyla	Cervidae	Praemegaceros_cazioti	0.273	0.332	Y	N	N
Cetartiodactyla	Cervidae	Sangamona_fugitiva	0.273	0.332	Y	N	N
Cetartiodactyla	Cervidae	Sinomegaceros_ordosianus	0.273	0.332	Y	N	N
Cetartiodactyla	Cervidae	Sinomegaceros_yabei	0.273	0.332	Y	N	N
Cetartiodactyla	Hippopotamidae	Hexaprotodon_sivalensis	0.25	0.332	Y	N	N
Cetartiodactyla	Hippopotamidae	Phanourios_minutes	0.25	0.332	Y	N	N
Cetartiodactyla	Suidae	Kolpochoerus_majus	0.889	0.332	Y	Y	Y
Cetartiodactyla	Suidae	Metridiochoerus_compactus	0.889	0.332	Y	Y	Y
Cetartiodactyla	Suidae	Sus_brachgnathus	0.889	0.332	Y	Y	Y
Cetartiodactyla	Tayassuidae	Catagonus_stenocephalus	0.75	0.332	Y	Y	Y
Cetartiodactyla	Tayassuidae	Muknalia_minima	0.75	0.332	Y	Y	Y
Cetartiodactyla	Tayassuidae	Mylohyus_nasutus	0.75	0.332	Y	Y	Y
Cetartiodactyla	Tayassuidae	Platygonus_compressus	0.75	0.332	Y	Y	Y
Chiroptera	Mormoopidae	Mormoops_magna	0	0.271	N	N	N
Chiroptera	Phyllostomidae	Artibeus_anthonyi	0.776	0.271	Y	Y	Y
Chiroptera	Phyllostomidae	Desmodus_draculae	0.776	0.271	N	N	N
Chiroptera	Phyllostomidae	Desmodus_puntajudensis	0.776	0.271	N	N	N
Chiroptera	Phyllostomidae	Desmodus_stocki	0.776	0.271	N	N	N
Chiroptera	Phyllostomidae	Phyllonycteris_major	0.776	0.271	Y	Y	Y
Chiroptera	Phyllostomidae	Phyllops_silvai	0.776	0.271	Y	Y	Y
Chiroptera	Pteropodidae	Pteropus_allenorum	0.936	0.271	Y	Y	Y
Chiroptera	Pteropodidae	Pteropus_coxi	0.936	0.271	Y	Y	Y
Cingulata	Dasypodidae	Dasypus_bellus	0.095	0.095	N	N	N
Cingulata	Dasypodidae	Eutatus_seguini	0.095	0.095	Y	Y	N
Cingulata	Dasypodidae	Propraopus_punctatus	0.095	0.095	N	N	N
Cingulata	Dasypodidae	Propraopus_sulcatus	0.095	0.095	N	N	N
Cingulata	Chlamyphoridae	Doedicurus_clavicaudatus	NA	0.095	Y	N	N
Cingulata	Chlamyphoridae	Glyptodon_clavipes	NA	0.095	Y	Y	N
Cingulata	Chlamyphoridae	Glyptodon_reticulatus	NA	0.095	Y	Y	N

Order	Family	Binomial name	$Prop_{frug}$ (Family-level)	$Prop_{frug}$ (Order-level)	L	D	C
Cingulata	Chlamyphoridae	Glyptotherium_cylindricum	NA	0.095	Y	N	N
Cingulata	Chlamyphoridae	Glyptotherium_floridanum	NA	0.095	Y	N	N
Cingulata	Chlamyphoridae	Hoplophorus_euphractus	NA	0.095	Y	N	N
Cingulata	Chlamyphoridae	Neosclerocalyptus_paskoenis	NA	0.095	Y	Y	N
Cingulata	Chlamyphoridae	Neuryurus_trabeculatus	NA	0.095	Y	N	N
Cingulata	Chlamyphoridae	Panochthus_tuberculatus	NA	0.095	Y	N	N
Cingulata	Pampatheriidae	Holmesina_occidentalis	NA	0.095	Y	N	N
Cingulata	Pampatheriidae	Holmesina_paulacoutoi	NA	0.095	Y	N	N
Cingulata	Pampatheriidae	Holmesina_septentrionalis	NA	0.095	Y	N	N
Cingulata	Pampatheriidae	Pampatherium_humboldti	NA	0.095	Y	N	N
Cingulata	Pampatheriidae	Pampatherium_typum	NA	0.095	Y	N	N
Cingulata	Incertae Sedis	Pachyarmatherium_brasiliense	NA	0.095	N	N	N
Diprotodontia	Diprotodontidae	Diprotodon_optatum	NA	0.452	Y	N	N
Diprotodontia	Diprotodontidae	Maokopia_ronaldi	NA	0.452	Y	N	N
Diprotodontia	Diprotodontidae	Palorchestes_azeal	NA	0.452	Y	N	N
Diprotodontia	Diprotodontidae	Zygomaturus_trilobus	NA	0.452	Y	N	N
Diprotodontia	Hypsiprymnodontidae	Propleopus_oscillans	1	0.452	N	N	N
Diprotodontia	Macropodidae	Macropus_ferragus	0.313	0.452	Y	Y	N
Diprotodontia	Macropodidae	Macropus_pearsoni	0.313	0.452	Y	Y	N
Diprotodontia	Macropodidae	Metasthenurus_newtonae	0.313	0.452	Y	Y	N
Diprotodontia	Macropodidae	Procoptodon_browneorum	0.313	0.452	Y	Y	N
Diprotodontia	Macropodidae	Procoptodon_gilli	0.313	0.452	Y	Y	N
Diprotodontia	Macropodidae	Procoptodon_goliah	0.313	0.452	Y	Y	N
Diprotodontia	Macropodidae	Procoptodon_oreas	0.313	0.452	Y	Y	N
Diprotodontia	Macropodidae	Procoptodon_rapha	0.313	0.452	Y	Y	N
Diprotodontia	Macropodidae	Protemnodon_anak	0.313	0.452	Y	Y	N
Diprotodontia	Macropodidae	Protemnodon_brehus	0.313	0.452	Y	Y	N
Diprotodontia	Macropodidae	Protemnodon_hopei	0.313	0.452	Y	Y	N
Diprotodontia	Macropodidae	Protemnodon_nombe	0.313	0.452	Y	Y	N
Diprotodontia	Macropodidae	Protemnodon_roechus	0.313	0.452	Y	Y	N
Diprotodontia	Macropodidae	Protemnodon_tumbuna	0.313	0.452	Y	Y	N
Diprotodontia	Macropodidae	Simosthenurus_maddocki	0.313	0.452	Y	Y	N
Diprotodontia	Macropodidae	Simosthenurus_occidentalis	0.313	0.452	Y	Y	N
Diprotodontia	Macropodidae	Simosthenurus_pales	0.313	0.452	Y	Y	N
Diprotodontia	Macropodidae	Sthenurus_andersoni	0.313	0.452	Y	Y	N
Diprotodontia	Macropodidae	Sthenurus_atlas	0.313	0.452	Y	Y	N
Diprotodontia	Macropodidae	Sthenurus_stirlingi	0.313	0.452	Y	Y	N
Diprotodontia	Macropodidae	Sthenurus_tindalei	0.313	0.452	Y	Y	N
Diprotodontia	Macropodidae	Thylogale_christenseni	0.313	0.452	Y	Y	N
Diprotodontia	Macropodidae	Troposodon_minor	0.313	0.452	Y	Y	N
Diprotodontia	Macropodidae	Wallabia_kitcheneri	0.313	0.452	Y	Y	N
Diprotodontia	Petauridae	Dactylopsila_kambuayai	0.182	0.452	N	N	N
Diprotodontia	Potoroidae	Borungaboodie_hatcheri	0	0.452	Y	N	N
Diprotodontia	Pseudocheiridae	Petauroides_ayamaruensis	0.778	0.452	Y	Y	Y
Diprotodontia	Thylacoleonidae	Thylacoleo_carnifex	NA	0.452	N	N	N
Diprotodontia	Vombatidae	Phascolonus_gigas	0	0.452	Y	N	N
Diprotodontia	Vombatidae	Vombatus_hacketti	0	0.452	Y	N	N
Diprotodontia	Vombatidae	Warendja_wakefieldi	0	0.452	Y	N	N
Eulipotyphla	Nesophontidae	Nesophontes_spA	0	0.02	N	N	N
Eulipotyphla	Soricidae	Nesiotites_hidalgo	0	0.02	N	N	N
Eulipotyphla	Soricidae	Nesiotites_similis	0	0.02	N	N	N
Lagomorpha	Leporidae	Aztlanolagus_agilis	0.065	0.043	Y	N	N

Order	Family	Binomial name	$Prop_{frug}$ (Family-level)	$Prop_{frug}$ (Order-level)	L	D	C
Lagomorpha	Ochotonidae	Ochotona_whartoni	0	0.043	Y	N	N
Litopterna	Macrauchenidae	Macrauchenia_patachonica	NA	NA	Y	Y	N
Litopterna	Macrauchenidae	Xenorhinotherium_bahiense	NA	NA	Y	Y	N
Litopterna	Proterotheriidae	Neolicaphrium_recens	NA	NA	Y	Y	N
Monotremata	Tachyglossidae	Megalibgwilia_ramsayi	0	0	N	N	N
Monotremata	Tachyglossidae	Zaglossus_hacketti	0	0	N	N	N
Notoungulata	Toxodontidae	Mixotoxodon_larensis	NA	NA	Y	Y	N
Notoungulata	Toxodontidae	Toxodon_platensis	NA	NA	Y	Y	N
Notoungulata	Toxodontidae	Trigonodops_lopesi	NA	NA	Y	Y	N
Peramelemorphia	Peramelidae	Peroryctes_spA	0.368	0.318	N	N	N
Peramelemorphia	Peramelidae	PeroryctinaeGen_spA	0.368	0.318	N	N	N
Perissodactyla	Equidae	Equus_francisci	0	0.438	Y	N	N
Perissodactyla	Equidae	Equus_hydruntinus	0	0.438	Y	N	N
Perissodactyla	Equidae	Equus_ovodovi	0	0.438	Y	N	N
Perissodactyla	Equidae	Hippidion_devillei	0	0.438	Y	Y	N
Perissodactyla	Equidae	Hippidion_principale	0	0.438	Y	Y	N
Perissodactyla	Rhinocerotidae	Coelodonta_antiquitatis	0.6	0.438	Y	Y	Y
Perissodactyla	Rhinocerotidae	Elasmotherium_sibiricum	0.6	0.438	Y	Y	Y
Perissodactyla	Rhinocerotidae	Stephanorhinus_hemitoechus	0.6	0.438	Y	Y	Y
Perissodactyla	Rhinocerotidae	Stephanorhinus_kirchbergensis	0.6	0.438	Y	Y	Y
Perissodactyla	Tapiridae	Tapirus_augustus	1	0.438	Y	Y	Y
Perissodactyla	Tapiridae	Tapirus_merriami	1	0.438	Y	Y	Y
Perissodactyla	Tapiridae	Tapirus_rondoniensis	1	0.438	Y	Y	Y
Perissodactyla	Tapiridae	Tapirus_veroensis	1	0.438	Y	Y	Y
Pholidota	Manidae	Manis_paleojavanica	0	0	N	N	N
Pilosa	Megalonychidae	Acratocnus_odontrigonus	1	0.2	Y	Y	Y
Pilosa	Megalonychidae	Acratocnus_ye	1	0.2	Y	Y	Y
Pilosa	Megalonychidae	Diabolotherium_nordenskioldi	1	0.2	Y	Y	Y
Pilosa	Megalonychidae	Megalocnus_rodens	1	0.2	Y	Y	Y
Pilosa	Megalonychidae	Megalocnus_zile	1	0.2	Y	Y	Y
Pilosa	Megalonychidae	Megalonyx_jeffersonii	1	0.2	Y	Y	Y
Pilosa	Megalonychidae	Neocnus_comes	1	0.2	Y	Y	Y
Pilosa	Megalonychidae	Neocnus_dousman	1	0.2	Y	Y	Y
Pilosa	Megalonychidae	Neocnus_toupiti	1	0.2	Y	Y	Y
Pilosa	Megalonychidae	Parocnus_browni	1	0.2	Y	Y	Y
Pilosa	Megalonychidae	Parocnus_serus	1	0.2	Y	Y	Y
Pilosa	Megatheriidae	Eremotherium_laurillardi	NA	0.2	Y	Y	N
Pilosa	Megatheriidae	Megatherium_americanum	NA	0.2	Y	Y	N
Pilosa	Megatheriidae	Megatherium_tarijense	NA	0.2	Y	Y	N
Pilosa	Mylodontidae	Catonyx_cuvieri	NA	0.2	Y	Y	N
Pilosa	Mylodontidae	Glossotherium_robustum	NA	0.2	Y	Y	N
Pilosa	Mylodontidae	Lestodon_armatus	NA	0.2	Y	Y	N
Pilosa	Mylodontidae	Mylodon_darwinii	NA	0.2	Y	Y	N
Pilosa	Mylodontidae	Paramylodon_harlani	NA	0.2	Y	Y	N
Pilosa	Mylodontidae	Scelidodon_chiliensis	NA	0.2	Y	Y	N
Pilosa	Mylodontidae	Scelidotherium_leptocephalum	NA	0.2	Y	Y	N
Pilosa	Mylodontidae	Valgipes_bucklandi	NA	0.2	Y	Y	N
Pilosa	Nothrotheridae	Nothrotheriops_shastensis	NA	0.2	Y	Y	N
Pilosa	Nothrotheridae	Nothrotherium_maquinense	NA	0.2	Y	Y	N
Primates	Archaeolemuridae	Archaeolemur_edwardsi	NA	0.793	Y	Y	Y
Primates	Archaeolemuridae	Archaeolemur_majori	NA	0.793	Y	Y	Y
Primates	Archaeolemuridae	Hadropithecus_stenognathus	NA	0.793	Y	Y	Y

Order	Family	Binomial name	Prop$_{frug}$ (Family-level)	Prop$_{frug}$ (Order-level)	L	D	C
Primates	Atelidae	Caipora_bambuiorum	1	0.793	Y	Y	Y
Primates	Atelidae	Protopithecus_brasiliensis	1	0.793	Y	Y	Y
Primates	Daubentoniidae	Daubentonia_robustus	0	0.793	Y	N	N
Primates	Hominidae	Homo_floresiensis	0.857	0.793	Y	Y	Y
Primates	Hominidae	Homo_neanderthalensis	0.857	0.793	N	N	N
Primates	Hominidae	Homo_spDenisova	0.857	0.793	Y	Y	Y
Primates	LeMuridae	Pachylemur_insignis	0.7	0.793	Y	Y	Y
Primates	LeMuridae	Pachylemur_jullyi	0.7	0.793	Y	Y	Y
Primates	Megaladapidae	Megaladapis_edwardsi	NA	0.793	Y	Y	Y
Primates	Megaladapidae	Megaladapis_grandidieri	NA	0.793	Y	Y	Y
Primates	Megaladapidae	Megaladapis_madagascariensis	NA	0.793	Y	Y	Y
Primates	Palaeopropithecidae	Archaeoindris_fontoynonti	1	0.793	Y	Y	Y
Primates	Palaeopropithecidae	Babakotia_radofilai	1	0.793	Y	Y	Y
Primates	Palaeopropithecidae	Mesopropithecus_globiceps	1	0.793	Y	Y	Y
Primates	Palaeopropithecidae	Mesopropithecus_pithecoides	1	0.793	Y	Y	Y
Primates	Palaeopropithecidae	Palaeopropithecus_maximus	1	0.793	Y	Y	Y
Primates	Pitheciidae	Antillothrix_bernensis	1	0.793	Y	Y	Y
Primates	Pitheciidae	Insulacebus_toussaintiana	1	0.793	Y	Y	Y
Proboscidea	Elephantidae	Elephas_antiquus	1	1	Y	Y	Y
Proboscidea	Elephantidae	Elephas_cypriotes	1	1	Y	Y	Y
Proboscidea	Elephantidae	Elephas_iolensis	1	1	Y	Y	Y
Proboscidea	Elephantidae	Elephas_mnaidriensis	1	1	Y	Y	Y
Proboscidea	Elephantidae	Elephas_namadicus	1	1	Y	Y	Y
Proboscidea	Elephantidae	Elephas_naumanii	1	1	Y	Y	Y
Proboscidea	Elephantidae	Elephas_tiliensis	1	1	Y	Y	Y
Proboscidea	Elephantidae	Mammuthus_columbi	1	1	Y	Y	Y
Proboscidea	Elephantidae	Mammuthus_exilis	1	1	Y	Y	Y
Proboscidea	Elephantidae	Mammuthus_primigenius	1	1	Y	Y	Y
Proboscidea	Gomphotheriidae	Cuvieronius_hyodon	NA	1	Y	Y	Y
Proboscidea	Gomphotheriidae	Notiomastodon_platensis	NA	1	Y	Y	Y
Proboscidea	Mammutidae	Mammut_americanum	NA	1	Y	Y	Y
Proboscidea	Stegodontidae	Stegodon_florensis	NA	1	Y	Y	Y
Proboscidea	Stegodontidae	Stegodon_orientalis	NA	1	Y	Y	Y
Proboscidea	Stegodontidae	Stegodon_trigonocephalus	NA	1	Y	Y	Y
Rodentia	Castoridae	Castoroides_ohioensis	0	0.394	Y	N	N
Rodentia	Caviidae	Neochoerus_aesopi	0.278	0.394	Y	N	N
Rodentia	Cricetidae	Antillomys_rayi	0.262	0.394	Y	N	N
Rodentia	Cricetidae	Megalomys_audreyae	0.262	0.394	Y	N	N
Rodentia	Cricetidae	Megalomys_georginae	0.262	0.394	Y	N	N
Rodentia	Cricetidae	Megaoryzomys_spA	0.262	0.394	Y	N	N
Rodentia	Cricetidae	Mesocricetus_rathgeberi	0.262	0.394	Y	N	N
Rodentia	Cricetidae	Microtus_henseli	0.262	0.394	Y	N	N
Rodentia	Cricetidae	Nesoryzomys_spB	0.262	0.394	Y	N	N
Rodentia	Cricetidae	Nesoryzomys_spC	0.262	0.394	Y	N	N
Rodentia	Cricetidae	Nesoryzomys_spD	0.262	0.394	Y	N	N
Rodentia	Cricetidae	Peromyscus_nesodytes	0.262	0.394	N	N	N
Rodentia	Cricetidae	Pliomys_lenki	0.262	0.394	Y	N	N
Rodentia	Echimyidae	Amblyrhiza_inundata	0.899	0.394	Y	Y	Y
Rodentia	Echimyidae	Geocapromys_spA	0.899	0.394	Y	Y	Y
Rodentia	Echimyidae	HexolobodontinaeGen_spA	0.899	0.394	Y	Y	Y
Rodentia	Echimyidae	Plagiodontia_spelaeum	0.899	0.394	Y	Y	Y
Rodentia	Echimyidae	Quemisia_gravis	0.899	0.394	Y	Y	Y

Order	Family	Binomial name	$Prop_{frug}$ (Family-level)	$Prop_{frug}$ (Order-level)	L	D	C
Rodentia	Echimyidae	Rhizoplagiodontia_lemkei	0.899	0.394	Y	Y	Y
Rodentia	Echimyidae	Xaymaca_fulvopulvis	0.899	0.394	Y	Y	Y
Rodentia	Gliridae	Eliomys_morpheus	0.857	0.394	Y	Y	Y
Rodentia	Hystricidae	Hystrix_kiangsenensis	1	0.394	Y	Y	Y
Rodentia	Hystricidae	Hystrix_refossa	1	0.394	Y	Y	Y
Rodentia	Muridae	Canariomys_bravoi	0.423	0.394	Y	Y	N
Rodentia	Muridae	Canariomys_tamarani	0.423	0.394	Y	Y	N
Rodentia	Muridae	Coryphomys_musseri	0.423	0.394	Y	Y	N
Rodentia	Muridae	Malpaisomys_insularis	0.423	0.394	Y	Y	N
Rodentia	Muridae	Papagomys_theodorverhoeveni	0.423	0.394	Y	Y	N
Rodentia	Muridae	Rattus_sanila	0.423	0.394	Y	Y	N
Rodentia	Muridae	Rhagamys_orthodon	0.423	0.394	Y	Y	N
Rodentia	Muridae	Solomys_spriggsarum	0.423	0.394	Y	Y	N
Rodentia	Muridae	Spelaeomys_florensis	0.423	0.394	Y	Y	N
Rodentia	Nesomyidae	Hypogeomys_australis	0.183	0.394	Y	N	N

Supplementary Table 8: **Principal component (PC) loadings for present-day bio-climatic variables.** The first three principal components were used as predictor variables in both the ordinary least squares and spatial autoregressive regression models.

Variable	PC1	PC2	PC3	PC4	PC5	PC6
**Global**						
Annual Precipitation	0.435	-0.279	0.69	-0.217	-0.458	-0.007
Precipitation Seasonality	-0.299	0.51	0.671	-0.059	0.444	-0.017
Precipitation of Driest Quarter	0.39	-0.428	0.148	0.53	0.602	-0.003
Mean Annual Temperature	0.341	0.532	-0.018	0.491	-0.269	0.536
Mean Temp. of Coldest Quarter	0.458	0.422	-0.121	0.052	-0.017	-0.771
Temperature Seasonality	-0.493	-0.133	0.194	0.652	-0.399	-0.344
Cumulative variance explained	0.522	0.835	0.907	0.969	1	1
**Afrotropics**						
Annual Precipitation	0.517	0.023	-0.028	0.704	0.485	-0.001
Precipitation Seasonality	-0.441	0.343	0.032	0.659	-0.501	0.041
Precipitation of Driest Quarter	0.465	-0.114	0.758	-0.003	-0.443	0.023
Mean annual temperature	-0.037	0.682	0.293	-0.161	0.257	-0.597
Mean temp. of Coldest Quarter	0.256	0.625	-0.092	-0.202	-0.012	0.703
Temperature Seasonality	-0.505	-0.115	0.574	0.052	0.502	0.384
Cumulative variance explained	0.5	0.834	0.92	0.974	1	1
**Neotropics**						
Annual Precipitation	0.43	0.118	-0.614	-0.582	0.294	-0.007
Precipitation Seasonality	-0.232	-0.624	-0.505	-0.042	-0.548	0.02
Precipitation of Driest Quarter	0.32	0.606	-0.204	0.243	-0.656	0.016
Mean Annual Temperature	0.456	-0.265	0.451	-0.338	-0.301	-0.56
Mean Temp. of Coldest Quarter	0.49	-0.284	0.247	-0.009	-0.084	0.782
Temperature seasonality	-0.46	0.282	0.25	-0.698	-0.294	0.272
Cumulative variance explained	0.575	0.838	0.956	0.989	1	1
**Indo-Australia**						
Annual Precipitation	-0.409	0.161	0.767	-0.026	-0.467	-0.01
Precipitation Seasonality	0.293	-0.571	0.564	0.245	0.459	-0.019
Precipitation of Driest Quarter	-0.388	0.489	0.023	0.587	0.514	0.001
Mean Annual Temperature	-0.382	-0.51	-0.253	0.436	-0.292	0.505
Mean Temp. of Coldest Quarter	-0.462	-0.367	-0.169	-0.006	0.016	-0.789
Temperature seasonality	0.487	0.121	-0.035	0.636	-0.471	-0.348
Cumulative variance explained	0.615	0.859	0.951	0.985	1	1

Supplementary Table 9: **Extinction probabilities of species of different IUCN Red List statuses over a 100 year period**. Probabilities inferred using Hoffmann *et al.*[1] and Di Marco *et al.*[2] datasets were derived using maximum likelihood rate estimates of the CTMC model (see equation 1 and 2 in Methods). Extinction probabilities of threatened categories from Davis *et al.*[6] are derived from IUCN Red List criteria[7]. Extinction rates for LC and NT categories in Davis *et al.*[6] were extrapolated from rates for threatened taxa by assuming an extinction rate increase exponentially with increasing severity in IUCN category (see Methods).

IUCN Red List category	Hoffmann *et al.* (2010) (All mammals)	Di Marco *et al.* (2014) (Carnivores + ungulates)	Davis *et al.* (2018) (Red List definitions and extrapolation)
LC	0.0001	0.0026	0.0017
NT	0.0041	0.0199	0.0141
VU	0.0139	0.0385	0.1000
EN	0.0548	0.0844	0.6723
CR	0.1802	0.2048	0.9990

Supplementary References

[1] Hoffmann, M. *et al.* The Impact of Conservation on the Status of the World's Vertebrates. *Science* **330**, 1503–1509 (2010).

[2] Di Marco, M. *et al.* A Retrospective Evaluation of the Global Decline of Carnivores and Ungulates. *Conservation Biology* **28**, 1109–1118 (2014).

[3] Cade, B. S. Model averaging and muddled multimodel inferences. *Ecology* **96**, 2370–2382 (2015).

[4] Faurby, S. *et al.* PHYLACINE1.2: The Phylogenetic Atlas of Mammal Macroecology. *Ecology* **99**, 2626 (2018).

[5] Kissling, W. D. *et al.* Establishing macroecological trait datasets: digitalization, extrapolation, and validation of diet preferences in terrestrial mammals worldwide. *Ecology and Evolution* **4**, 2913–2930 (2014).

[6] Davis, M., Faurby, S. & Svenning, J. C. Mammal diversity will take millions of years to recover from the current biodiversity crisis. *Proceedings of the National Academy of Sciences of the United States of America* **115**, 11262–11267 (2018).

[7] Mooers, A. Ø., Faith, D. P. & Maddison, W. P. Converting Endangered Species Categories to Probabilities of Extinction for Phylogenetic Conservation Prioritization. *PLoS ONE* **3**, e3700 (2008).
