# Supplementary material for: Frugivore-fruit size relationships between palms and mammals reveal past and future defaunation impacts
Source: Nat Commun. 2020 Sep 29;11:4904. doi: 10.1038/s41467-020-18530-5 (PMC7524719; doi:10.1038/s41467-020-18530-5)
Supplement: Supplementary file 3 — Reporting Summary [file 41467_2020_18530_MOESM3_ESM.pdf]

nature research

Corresponding author(s): Lim, Jun Ying

Last updated by author(s): Aug 11, 2020

Reporting Summary

Nature Research wishes to improve the reproducibility of the work that we publish. This form provides structure for consistency and transparency in reporting. For further information on Nature Research policies, see our Editorial Policies and the Editorial Policy Checklist.

Statistics

For all statistical analyses, confirm that the following items are present in the figure legend, table legend, main text, or Methods section.

n/a	Confirmed	
☐	☒	The exact sample size (*n*) for each experimental group/condition, given as a discrete number and unit of measurement
☐	☒	A statement on whether measurements were taken from distinct samples or whether the same sample was measured repeatedly
☐	☒	The statistical test(s) used AND whether they are one- or two-sided<br>*Only common tests should be described solely by name; describe more complex techniques in the Methods section.*
☐	☒	A description of all covariates tested
☐	☒	A description of any assumptions or corrections, such as tests of normality and adjustment for multiple comparisons
☐	☒	A full description of the statistical parameters including central tendency (e.g. means) or other basic estimates (e.g. regression coefficient) AND variation (e.g. standard deviation) or associated estimates of uncertainty (e.g. confidence intervals)
☐	☒	For null hypothesis testing, the test statistic (e.g. *F*, *t*, *r*) with confidence intervals, effect sizes, degrees of freedom and *P* value noted<br>*Give P values as exact values whenever suitable.*
☒	☐	For Bayesian analysis, information on the choice of priors and Markov chain Monte Carlo settings
☒	☐	For hierarchical and complex designs, identification of the appropriate level for tests and full reporting of outcomes
☐	☒	Estimates of effect sizes (e.g. Cohen's *d*, Pearson's *r*), indicating how they were calculated

Our web collection on statistics for biologists contains articles on many of the points above.

Software and code

Policy information about availability of computer code

Data collection	No software was used in data collection
Data analysis	Statistical analyses were performed using custom R scripts which were run on R version 3.5.1 and used the following packages and their dependencies: \`stringr\` (v1.4.0), \`scales\` (v1.0.0), \`cowplot\` (v1.0.0), \`reshape2\` (v1.4.3), \`MuMIn\` (v1.43.6), \`viridis\` (v0.5.1), \`wesanderson\` (v0.3.6), \`RColorBrewer\` (v1.1-2), \`ggplot2\` (v3.2.1), \`rgdal\` (v1.4-4), \`sp\` (v1.3-1), \`relaimpo\` (v2.2-3), \`car\` (v3.0-2), \`care\` (v1.1.10), \`spatialreg\` (v1.1-3), \`spdep\` (v1.1-2). Data and scripts are publicly available in a Dryad digital repository (doi:10.5061/dryad.6hdr7sqwt)

For manuscripts utilizing custom algorithms or software that are central to the research but not yet described in published literature, software must be made available to editors and reviewers. We strongly encourage code deposition in a community repository (e.g. GitHub). See the Nature Research guidelines for submitting code & software for further information.

Data

Policy information about availability of data

All manuscripts must include a data availability statement. This statement should provide the following information, where applicable:

- Accession codes, unique identifiers, or web links for publicly available datasets
- A list of figures that have associated raw data
- A description of any restrictions on data availability

Palm trait data are openly available (Kissling et al. 2019 Scientific Data, 6, 178.). Palm distribution data was obtained from the Kew World Checklist of Selected Plant Species (http://wcsp.science.kew.org/home.do; downloaded June 2015). Mammal distribution, body size data and IUCN Red List statuses were obtained from the openly available PHYLACINE dataset (v1.2; Faurby et al. 2018, Ecology, 99, 2626–2626). Associated data derived from such datasets and custom analysis scripts are stored in a publicly available Dryad digital repository (doi:10.5061/dryad.6hdr7sqwt).

Field-specific reporting

Please select the one below that is the best fit for your research. If you are not sure, read the appropriate sections before making your selection.

☐ Life sciences ☐ Behavioural & social sciences ☒ Ecological, evolutionary & environmental sciences

For a reference copy of the document with all sections, see nature.com/documents/nr-reporting-summary-flat.pdf

Ecological, evolutionary & environmental sciences study design

All studies must disclose on these points even when the disclosure is negative.

Study description	We examined the relationship between the body size of mammalian frugivore assemblages (present-day and past) and present-day fruit size of palm assemblages at both global and regional scales using a model-averaging approach. We additionally project changes in fruit size given potential changes in body size in future mammalian frugivore assemblages under probabilistic simulations of extinction.
Research sample	Palms (family Arecaceae) were selected as they represent a key component of tropical ecosystems, are almost entirely animal-dispersed, and because distribution and fruit size data was available for almost all species globally. Mammals were chosen as many palm fruits (especially species with large fruit) rely on mammalian frugivores for seed dispersal. We calculated the maximum (95th percentile) and median palm fruit size and the maximum and median body size of mammalian frugivores at the scale of botanical countries (i.e., level-3 Taxonomic Databases Working Group geographic units). Mammal species were chosen for inclusion in our analysis based on their reported degree of frugivory or herbivory in the MammalDiet database (Kissling et al. 2014, Ecology & Evolution), as well as the PHYLACINE database (for extinct species)(Faurby et al. 2018, Ecology).
Sampling strategy	No statistical methods were used to predetermine sample size. Sampling units (level-3 units or botanical countries)were based on standardized geographic units as defined by the Taxonomic Databases Working Group. Botanical countries represented the most fine-scale geographic units for which reliable and expert-curated geographic distribution data for palms were also available. Sample sizes (Global, n = 129; Afrotropics, n = 51; Neotropics, n = 42; Indo-Australia, n = 36) were adequate for the number of variables used in our statistical models (minimum = 1, maximum = 6).
Data collection	Data used in our study are openly available and were downloaded from online repositories. The palm trait data used in this study (Kissling et al. 2019 Scientific Data, 6, 178.) was downloaded from a Dryad digital repository (doi:10.5061/dryad.ts45225). Palm distribution data was obtained from the Kew World Checklist of Selected Plant Species (http://wcsp.science.kew.org/home.do; downloaded June 2015). Mammal range maps, body size data and IUCN Red List statuses were obtained from the openly available PHYLACINE dataset (v1.2; Faurby et al. 2018, Ecology, 99, 2626; Dryad digital repository doi:10.5061/dryad.bp26v20). Mammal range maps were converted into checklists at the botanical-country scale by overlaying range maps with botanical country polygons using various functions in R. We determined which extant species were frugivores using information in the MammalDiet dataset (Dryad digital repository doi:10.5061/dryad.6cd0v). The diet of extinct species was determined using a combination of information from the MammalDiet dataset, PHYLACINE dataset as well as information obtained through a wider research literature of extinct taxa.
Timing and spatial scale	Data are global in extent. Body size and fruit size were determined at the spatial grain of botanical countries (i.e., level-3 Taxonomic Databases Working Group geographic units) as palm distribution data was only available at this spatial resolution. The "current" faunal scenario was based on present-day mammal diversity and distributions correct as of 2016 (IUCN 2016 v3), whereas the "present-natural" faunal scenario was based on Pleistocene mammal diversity and distributions at the Last Integlacial, ca. 130,000 years ago (as compiled in Faurby et al., 2018).
Data exclusions	Data exclusion criteria were pre-defined. We only focused on mammalian frugivore and palm assemblages within the Neotropics, Afrotropics and Indo-Australia as they possess distinct faunas and represented biogeographic regions that had sufficient and more-or-less equal sample size. We excluded countries in other biogeographic regions as they either have depauperate palm floras or are so physically isolated that they do not represent appropriate points of comparison with other regions. We additionally only included botanical countries for which extant mammalian frugivores are present in the database.
Reproducibility	Steps required to reproduce our study are described within our manuscript. In addition, all data and code to reproduce the analyses we performed (including figures used in the manuscript) is freely available and can be downloaded from a Dryad digital respository (doi:10.5061/dryad.6hdr7sqwt) .
Randomization	Data used in this study are derived from published datasets hence randomization of data is not applicable.
Blinding	Not applicable. Data used in this study are derived from published datasets. Data collection in that sense is blind to the hypotheses tested in this study.

Did the study involve field work? ☐ Yes ☒ No

Reporting for specific materials, systems and methods

We require information from authors about some types of materials, experimental systems and methods used in many studies. Here, indicate whether each material, system or method listed is relevant to your study. If you are not sure if a list item applies to your research, read the appropriate section before selecting a response.

Materials & experimental systems

n/a	Involved in the study
[x]	[ ] Antibodies
[x]	[ ] Eukaryotic cell lines
[x]	[ ] Palaeontology and archaeology
[x]	[ ] Animals and other organisms
[x]	[ ] Human research participants
[x]	[ ] Clinical data
[x]	[ ] Dual use research of concern

Methods

n/a	Involved in the study
[x]	[ ] ChIP-seq
[x]	[ ] Flow cytometry
[x]	[ ] MRI-based neuroimaging
